# Optimizing management of stage IV EGFR mutant non‐small cell lung cancer in Asia: An expert opinion

**DOI:** 10.1002/ijc.35512

**Published:** 2025-06-14

**Authors:** Gee‐Chen Chang, Akhil Kapoor, Chee Khoon Lee, Chunxia Su, Daniel Chan, Guia Elena Imelda Ladrera, Hye Ryun Kim, Mostafa Aziz Sumon, Moushumi Suryavanshi, Sita Andarini, Tatsuya Yoshida, Thanyanan Reungwetwattana, Tuan Khoi Nguyen, Pei Jye Voon

**Affiliations:** ^1^ Department of Internal Medicine, Division of Pulmonary Medicine Chung Shan Medical University Hospital Taichung Taiwan; ^2^ School of Medicine and Institute of Medicine Chung Shan Medical University Taichung Taiwan; ^3^ Institute of Biomedical Sciences National Chung Hsing University Taichung Taiwan; ^4^ Department of Medical Oncology, Homi Bhabha Cancer Hospital & Mahamana Pandit Madanmohan Malaviya Cancer Centre, Tata Memorial Centre Homi Bhabha National Institute Varanasi India; ^5^ Department of Medical Oncology Cancer Care Centre, St George Hospital Sydney Australia; ^6^ Medical Oncology Department Shanghai Pulmonary Hospital Shanghai China; ^7^ Icon Cancer Centre Singapore Singapore; ^8^ Medical Oncology Lung Centre of the Philippines Manilla Philippines; ^9^ Division of Medical Oncology, Yonsei Cancer Center Yonsei University College of Medicine Seoul South Korea; ^10^ Department of Radiation Oncology Kurmitola General Hospital Dhaka Bangladesh; ^11^ Department of Molecular Biology & Department of Pathology Amrita Institute of Medical Sciences and Research Centre Faridabad India; ^12^ Department of Pulmonology and Respiratory Medicine, Faculty of Medicine University of Indonesia—Persahabatan Hospital Jakarta Indonesia; ^13^ Department of Thoracic Oncology/Experimental Therapeutics National Cancer Center Hospital Tokyo Japan; ^14^ Division of Medical Oncology, Department of Medicine, Faculty of Medicine Ramathibodi Hospital Mahidol University Bangkok Thailand; ^15^ Department of Internal Medicine 1 Hochiminh Oncology Hospitals Ho Chi Minh Vietnam; ^16^ Department of Radiotherapy, Oncology and Palliative Care Hospital Umum Sarawak Kuching Malaysia

**Keywords:** Asian exert opinion, EGFR positive NSCLC, EGFRm NSCLC, NSCLC in Asia

## Abstract

Lung cancer is Asia's most prevalent cancer, accounting for the highest global patient share. A significant number of non‐small cell lung cancer (NSCLC) patients in Asia exhibit mutations in the epidermal growth factor receptor (EGFR). Although clinical outcomes are improving with newer therapies, challenges persist in the effective management of EGFR mutant (EGFRm) NSCLC. Given the substantial disease burden, understanding the current diagnostic and treatment patterns for EGFRm NSCLC from an Asian perspective is essential. This expert opinion presents recommendations from Asian experts on molecular testing and treatment of first‐line and second‐line EGFRm NSCLC. The recommendations aim to optimize patient outcomes by providing a comprehensive approach to diagnosis and management, considering the high prevalence of EGFR mutations in the Asian population. The experts discussed and recommended approaches for optimal management of EGFRm NSCLC. Next‐generation sequencing (NGS) testing is recommended to be included in the reimbursement scheme, and the turnaround time of testing should be shortened, considering the high burden of the disease in Asia. The panel recommended careful selection of patients for osimertinib+chemotherapy or lazertinib+amivantamab based on safety and efficacy profile, patient age, and disease status. While the panel agreed that osimertinib+chemotherapy is acceptable for these patients, dose adjustment and careful patient selection are recommended to optimize safety outcomes. For lazertinib+amivantamab, measures to mitigate adverse events such as the use of pre‐medication with steroids, prophylactic anticoagulants, and dose modification are recommended. For patients progressing on one of the combination regimens, experts recommended repeat NGS testing and continued treatment with chemotherapy.

## What's New?

Emerging strategies for the treatment of advanced non‐small cell lung cancer (NSCLC) featuring epidermal growth factor receptor (EGFR) mutations are helping improve patient outcomes. Which strategies are optimal to effectively manage EGFR‐mutant NSCLC, however, remains unclear. This study gathered insight on the diagnosis and treatment of advanced EGFR‐mutant NSCLC from a panel of lung cancer experts in Asia, where the lung cancer burden is high. Notably, the panel recommended next‐generation sequencing for identifying patients for EGFR TKI treatment and favored osimertinib plus chemotherapy over lazertinib plus amivantamab. The findings cast light on current diagnostic and treatment patterns for advanced EGFRm NSCLC.

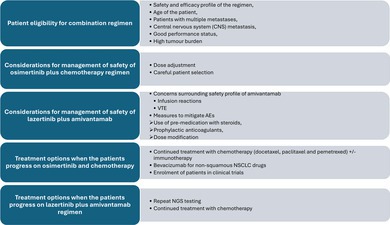

**Abbreviations**
ADCs: Antibody‐Drug Conjugates
AEs: Adverse Events
ALK: Anaplastic Lymphoma Kinase
ctDNA: Circulating Tumour DNA
EGFR: Epidermal Growth Factor Receptor
EGFRm: Epidermal Growth Factor Receptor Mutant
ESMO: European Society For Medical Oncology
IHC: Immunohistochemistry
IV: Intravenous
MET: Mesenchymal Epithelial Transition
NGS: Next‐Generation Sequencing
NSCLC: Non‐Small Cell Lung Cancer
NTRK: Neurotrophic Tyrosine Receptor Kinase
OS: Overall Survival
PCR: Polymerase Chain Reaction
PD‐L1: Programmed Death‐Ligand 1
PIK3CA: Phosphatidylinositol‐4,5‐Bisphosphate 3‐Kinase Catalytic Subunit Alpha
SC: Subcutaneous
TKI: Tyrosine Kinase Inhibitors
VTE: Venous Thromboembolism

## 1 INTRODUCTION

Lung cancer is the most prevalent cancer subtype in Asia, accounting for approximately 20% of all cancer cases in the region.^1^ It comprises 30%–35% of the global lung cancer burden, with about 85% of cases being non‐small cell lung cancer (NSCLC).^2^ Family history and genetic susceptibility are significant factors in the development of lung cancer, with substantial NSCLC cases, particularly those who are non‐smokers, linked to genetic mutations.^3^

Tobacco smoking is the most prominent modifiable risk factor for lung cancer, contributing to nearly 85% of lung cancer‐related deaths. However, there is a rising incidence of lung cancer among non‐smokers. In Taiwan, more than 53% of all NSCLC cases occur in individuals who have never smoked. This trend points to other risk factors beyond smoking, such as indoor coal burning, cooking fumes, and infections like tuberculosis.^4^, ^5^

In the Asian population, epidermal growth factor receptor (EGFR) mutation is the most common genetic alteration, found in approximately 40%–50% of all NSCLC patients, and as high as 75%–80% among never‐smoking Asians.^3^, ^4^ EGFR mutations correlate with sensitivity to EGFR tyrosine kinase inhibitors (TKIs), highlighting the importance of accurate mutation detection for personalized cancer therapy. Various methods are used to detect EGFR mutations in tumours and blood, including polymerase chain reaction (PCR)‐based, mass spectrometry‐based, and next‐generation sequencing (NGS)‐based techniques. Each method offers distinct advantages depending on the clinical scenario, sample type, and mutations of interest.^6^, ^7^

According to the European Society for Medical Oncology (ESMO) pan‐Asia guidelines for the management of NSCLC, patients with tumours harbouring a sensitizing EGFR mutation should receive any first‐line EGFR TKIs, including osimertinib, erlotinib, gefitinib, or afatinib, and for those experiencing disease progression, osimertinib is recommended following a liquid biopsy or tissue rebiopsy in those with acquired T790M mutation.^8^

The development of EGFR‐TKIs for NSCLC with EGFR mutations has advanced through three generations. Initially, first‐generation TKIs achieved a 5‐year overall survival (OS) of 14.6% (95% CI 9.7–21.9) for stage III/stage IV EGFR‐mutant (EGFRm) NSCLC.^9^ Subsequent development of second‐ and third‐generation TKIs improved the 5‐year OS rate to 28% (95% CI 22.1–35.7).^10^

Osimertinib is a third‐generation, mutant‐selective, irreversible inhibitor that selectively inhibits both EGFR‐TKI–sensitizing and EGFR p.Thr790Met (T790M) resistance mutations. The phase 3 FLAURA trial demonstrated that patients with previously untreated advanced NSCLC with an EGFR mutation had longer overall survival when treated with osimertinib monotherapy compared to those receiving other EGFR‐TKIs (38.6 vs. 31.8 months, respectively). Therefore, osimertinib is considered the preferred EGFR TKI according to various treatment guidelines.^8^, ^11^, ^12^ Despite the improved outcomes in the first‐line setting, most patients eventually experience disease progression due to acquired resistance mutations. Consequently, the combination of osimertinib and platinum‐based chemotherapy was explored in the phase 3 FLAURA2 trial for first‐line advanced EGFRm NSCLC.^13^

Amivantamab is a bispecific antibody that targets the EGFR and Mesenchymal Epithelial Transition (MET) receptors to inhibit ligand binding, promote downregulation of cell surface receptors, and induce Fc‐dependent trogocytosis and antibody‐dependent cellular cytotoxicity. By extracellular binding, amivantamab provides complementary action to EGFR‐TKIs, and the combination has shown superior outcomes compared to monotherapy.^14^ Lazertinib, a third‐generation EGFR‐TKI, inhibits EGFR mutations including classic cases (exon 19 deletion and L858R exon 21‐point mutation) and EGFR T790M resistance mutations while sparing wild‐type EGFR cells.^15^ The phase 3 MARIPOSA trial assessed the combination of amivantamab and lazertinib to determine its efficacy in the first‐line advanced EGFRm NSCLC population. The MARIPOSA study hypothesized that osimertinib resistance, seen in patients progressing on first‐line osimertinib, is primarily dependent on the C797S mutation of the EGFR gene and MET amplification.^16^

MET amplification is a common acquired resistance mechanism contributing to osimertinib resistance.^17^ According to the current treatment guidelines, patients who progress on first‐line treatment should undergo repeat genetic testing.^12^ However, only a few actionable resistance mechanisms are often identified, which limits treatment options primarily to chemotherapy and selected anti‐cancer agents, such as the regimen used in the MARIPOSA‐2 trial (amivantamab in combination with chemotherapy, with or without lazertinib).^8^, ^18^, ^19^

To address resistance mechanisms, new combination strategies are being explored, integrating EGFR TKIs with antibody‐drug conjugates (ADCs), bispecific antibodies, or MET inhibitors. ADCs, which include a cytotoxic payload such as topoisomerase inhibitors, have shown promising outcomes in early clinical data.^20^, ^21^

Given the emerging evidence for combined strategies involving third‐generation EGFR‐TKI agents and their potential benefit for the Asian population, where the disease burden is high, it is crucial to understand current diagnostic and treatment patterns for advanced EGFRm NSCLC from an Asian perspective. To this end, we assembled a panel of lung cancer experts from various Asian countries to gather insights and develop expert opinions for the diagnosis and treatment of advanced EGFRm NSCLC.

## 2 METHODS

The committee meeting comprised two components: a pre‐meeting survey followed by a virtual expert meeting that included discussions and voting questions.

The 14‐member scientific panel consisted of oncologists (11/14), pulmonologists (02/14), and a pathologist (01/14) from Australia, Bangladesh, China, India, Indonesia, Japan, Malaysia, the Philippines, Singapore, South Korea, Taiwan, Thailand, and Vietnam. Participants were invited based on their scientific interests, merit, and involvement in relevant research.

### 2.1 Pre‐meeting Survey

A 17‐question survey was developed to capture the opinions of participating experts regarding the diagnosis and treatment of stage IV EGFRm NSCLC. The invitation to the survey outlined the study's aims and objectives, allowing clinicians the opportunity to accept or decline participation. All clinicians voluntarily agreed to participate in the survey. The survey results were analysed, and a summary was developed to facilitate discussion during the virtual expert meeting held in June 2024.

### 2.2 Expert Meeting

All 14 panel members attended the meeting on June 29, 2024, to discuss the following topics:

- Interpretation of recent data regarding diagnosis and treatment for EGFR‐mutated advanced metastatic non‐small lung cancer
- The potential impact of these data on the diagnosis and treatment of EGFR‐mutated NSCLC in the first and second lines.

Specific discussion questions were framed to facilitate structured discussions, and voting questions were included to collect the panel's opinions.

## 3 RESULTS AND DISCUSSION

Answers to all pre‐meeting survey questions and voting questions are summarized in supplementary material.

### 3.1 Personalized treatment for stage IV NSCLC in the first‐line

#### 3.1.1 How to maximize next‐generation sequencing (NGS) testing for the patients of stage IV NSCLC?

*Statement*: NGS testing is recommended to be included in the reimbursement scheme, and the turnaround time of testing should be shortened.

**FIGURE 1:**
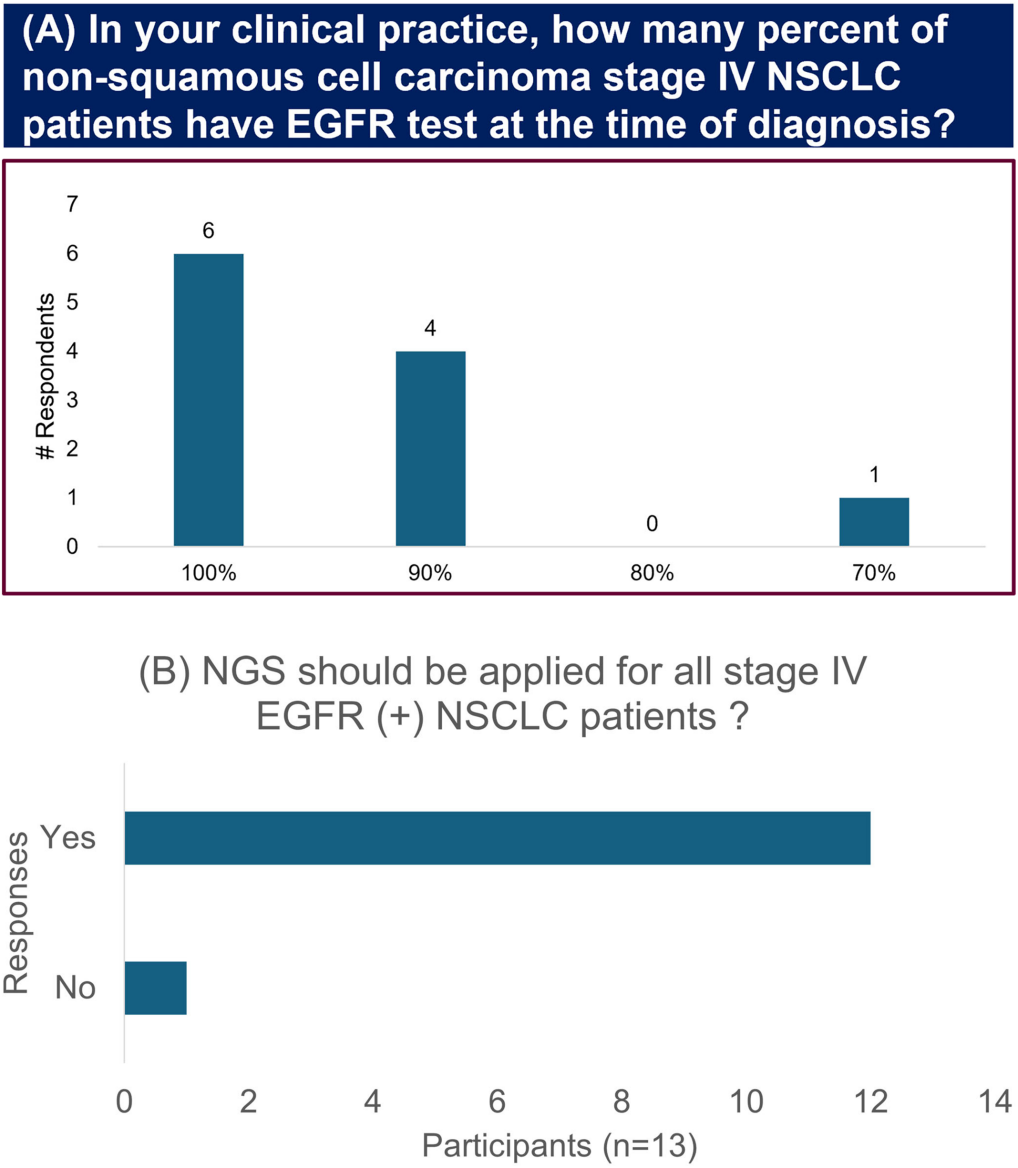
Preference for NGS testing among participating experts. (A) Pre‐meeting survey: Frequency of EGFR testing. (B) Voting: Preference for NGS testing. EGFR, Epidermal growth factor receptor; NGS, Next generation sequencing; NSCLC, Non‐small cell lung cancer.

In the pre‐meeting survey, all panel members estimated that between 50% and 100% of stage IV NSCLC patients have an EGFR mutation test (Figure 1A). Additionally, 92% of participants advocated for the application of NGS testing for all patients (Figure 1B).

Given the mutations affecting EGFR, parallel testing through NGS is preferred over single‐gene testing. NGS testing can be conducted before initiating treatment, during treatment, and after treatment resistance has developed. High‐throughput NGS testing allows for reliable and rapid identification of prevalent and well‐defined genetic aberrations in cancer patients, extending beyond specific hotspot mutation loci.^22^ Multigene NGS testing provides treating physicians with a comprehensive molecular profile, aiding in determining responsiveness to targeted drug therapies.^23^ Clinical trials have demonstrated that NGS testing can improve survival outcomes.^24^, ^25^, ^26^ However, in Asian countries, the use of NGS testing is inconsistent due to factors like cost, turnaround time, insurance coverage, and patient and provider education. Table 1 summarizes the current availability of NGS testing and its insurance reimbursement status across Asian countries. Due to inconsistent availability and high out‐of‐pocket costs, which often arise from the lack of insurance coverage, patients frequently opt for PCR or single‐gene testing as more affordable and quicker alternatives. This aligns with an Asian expert consensus on biomarker testing in NSCLC, which recommends PCR testing for stage II or higher non‐metastatic NSCLC.^27^

**TABLE 1:** Current status of genetic tests in metastatic non‐squamous NSCLC in Asian countries (as of 28th October 2024).

	EGFR mutation rate in non‐squamous NSCLC	EGFR PCR mutation test use Y/N, (%)	EGFR PCR mutation test reimbursement	Multiple × PCR test use Y/N, (%)	Multiplex PCR test reimbursement	NGS test use Y/N, (%)	NGS test reimbursement	Liquid biopsy use Y/N, (%)	Liquid biopsy reimbursement
Australia	15%	Yes	Yes	Yes	Yes	Yes	Yes	Yes	No
Bangladesh	25%	Yes, 20%	No	No	No	Yes, 60%	No	Yes, 20%	No
China	50%	80%	No	60%		No	No	Yes, 30%	No
India	39%–40%	Yes, 65%	Mostly no (70%)	Yes	Mostly no (70%)	Yes, 35%	Mostly no (70%)	Yes, mainly use for detection of T790M mutation	Mostly no (70%)
Indonesia	55%–60%	Yes, 80%	Yes (reimbursed by Universal Health Coverage)	No	No	Yes, <5%	No	Yes, mainly use for detection of T790M mutation; 10%	No. Partial reimbursement under industry sponsorship)
Japan	40%–50%	Yes, 10%	Yes	Yes, 30%	Yes	Yes, 60%	Yes	Yes, mainly use for detection of T790M mutation	Yes
Malaysia	45%–50%	Yes, 85%	Yes	No	No	Yes, 15%	No (at patient's own cost/private insurance coverage)	Yes, mainly use for detection of T790M mutation	Yes (Under industry sponsorship).
Philippines	70%	Yes 100%	No	No	No	Yes, <5%	No	Yes, <3%	No
Singapore	40% (all NSCLC); 60% Nonsquamous NSCLC	Yes, 80%	Yes	Yes, 50%	Yes	Yes, 30% (public) 80% (private)	NO (only private insurance)	Yes, mainly use for detection of T790M mutation and MET amp; 10%	No unless private insurance.
South Korea	30%–40%	Yes	Yes	Yes	No	Yes	Yes, 50%	Yes	No for G360, FMI, yes for cobas EGFR test
Taiwan	50%–55%	Yes, 80%	Yes	Yes, 10%	No	Yes, 30%	Yes, partially (20–30% of total cost) After EGFR PCR (−) NGS firstàpartially pay in EGFR wild type	Yes, <5%	No
Thailand	55%–65%	Yes, majority of testing.	It is partial reimbursement	Yes, <10%	Yes, It is partial reimbursement for CSMBS only.	Yes, <10%	Yes, It is partial reimbursement for CSMBS only.	Yes, mostly for T790M detection after 1st or 2nd gen EGFR TKI	Only liquid T790M testing is partial reimbursement for CSMBS only.
Vietnam	40%–50%	Yes, majority of testing.	It is partial reimbursement.	No	No	Yes, <50%	No	Yes, mostly for T790M detection after 1st or 2nd gen EGFR TKI or in case tissue biopsy could not be done.	Yes. Only for PCR. No reimbursement for liquid NGS testing.

Abbreviations: EGFR TKI, epidermal growth factor receptor tyrosine kinase inhibitor; NGS, next generation sequencing; n, no; NSCLC, non‐small cell lung cancer; PCR, polymerase chain reaction; y, yes.

To improve NGS testing affordability, the expert panel recommended the following solutions: include NGS testing in insurance reimbursement policies, mandate NGS testing in clinical trials, foster collaborations between the pharmaceutical and diagnostic industries to offer NGS testing at reduced rates, and educate healthcare providers. The panel also emphasized the importance of reducing the turnaround time for NGS test results. Finally, an exclusionary approach for NGS testing may be applied (starting from EGFR, Anaplastic lymphoma kinase (ALK) immunohistochemistry (IHC), and Programmed Death‐Ligand 1 (PD‐L1) then if negative then limited multigene panel testing either by NGS or other techniques may be considered).

#### 3.1.2 Which patients' group should be consulted about combination regimens including osimertinib plus chemotherapy or lazertinib plus amivantamab?

*Statement*: Multiple factors should be considered prior to the selection of patients for either monotherapy or one of the combination regimens such as the safety and efficacy profile of the regimen, age of the patient, patients with multiple metastases, central nervous system (CNS) metastasis, good performance status, and high tumour burden. Currently, there are no head‐to‐head comparison data to evaluate the efficacy and safety of osimertinib plus chemotherapy vs. Lazertinib plus amivantamab as first‐line treatment for advanced EGFR mutation NSCLC.

The choice of first‐line treatment depends on multiple factors, including the stage of the disease, tumor histology, molecular profile, safety and efficacy of the proposed treatments, age of the patient, comorbidities, and the patient's treatment preferences.^12^, ^28^

Osimertinib is the preferred first‐line treatment for patients with EGFRm advanced NSCLC.^2^ Despite its benefits, patients often experience disease progression, necessitating a combination approach, especially in patients with poor prognostic factors. However, despite the improved efficacy, adding more drugs in the first‐line treatment can lead to poorer safety outcomes, as seen in the FLAURA2 trial, where 64% of patients in the combination arm experienced grade 3 or higher adverse effects compared to 27% in the monotherapy arm.^13^ Similarly, in the MARIPOSA trial, despite the improved efficacy with the combination regimen, more adverse events (AEs) were reported in the combination arm, as compared to the monotherapy arm. Further, diverse AEs were reported which can be distinctively attributed to the various components of the combination (related to EGFR inhibition and related to MET inhibition).^16^ Therefore, it is crucial to determine which patients should receive monotherapy and which should receive a combination regimen. Patients typically present with multiple risk factors, and these should be considered before selecting the therapy.

**FIGURE 2:**
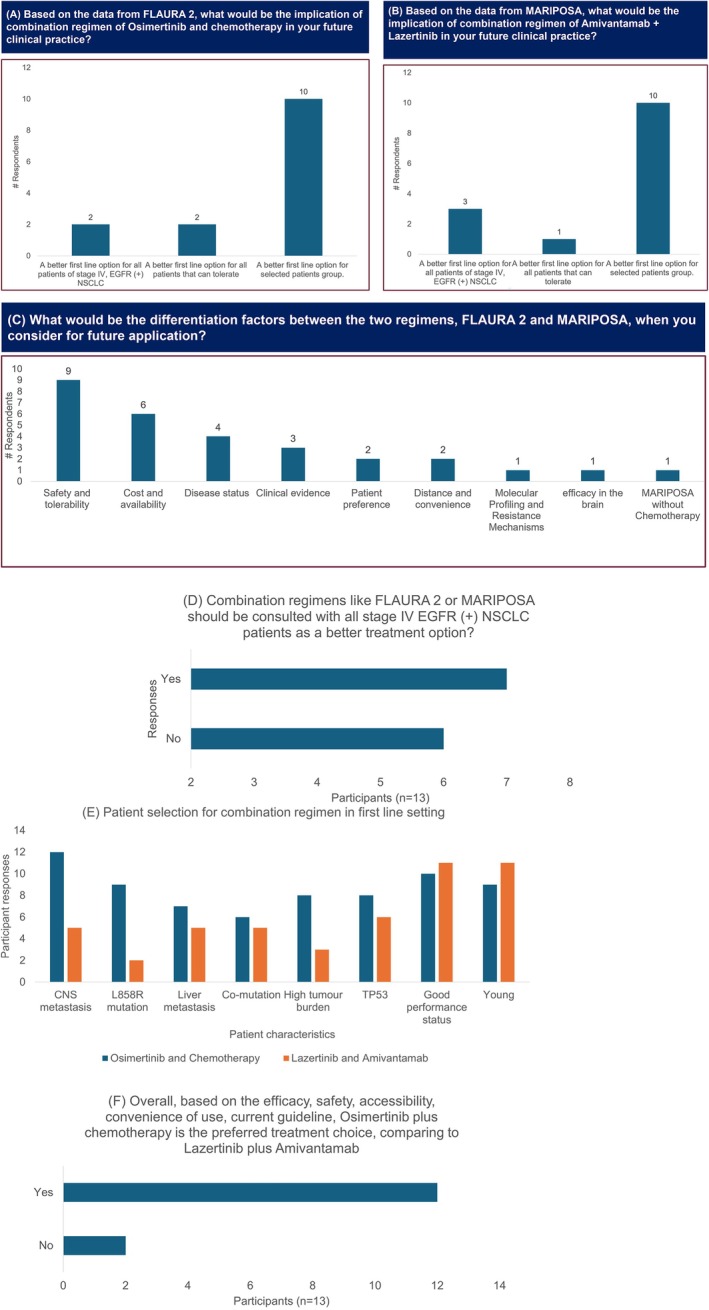
Patient selection criteria for first line therapy. (A) Pre‐meeting survey: Implications of using Osimertinib + chemotherapy regimen in clinical practice. (B) Pre‐meeting survey: Implications of using Lazertinib + amivantamab regimen in clinical practice. (C) Pre‐meeting survey: Differentiating factor between Osimertinib + chemotherapy and Lazertinib + amivantamab regimens. (D) Voting: Preference for consultation regarding combination regimens. (E) Voting: Patient selection for Osimertinib + chemotherapy and Lazertinib + amivantamab regimens. (F) Voting: Preferred therapy based on available evidence. CNS, Central nervous system; EGFR, Epidermal growth factor receptor; NSCLC, Non‐small cell lung cancer.

In the pre‐meeting survey, 10/14 participants responded that osimertinib plus chemotherapy and lazertinib plus amivantamab regimen are better first‐line therapy options for selected patients. During the survey and the discussion, experts elaborated on the selection criteria for these regimens, including the presence of CNS metastases, L858R mutations, age of patients, tumour burden, and the presence of other metastases (Figure 2A,B). Differentiating factors of the two regimens were also identified, which include the safety and tolerability profiles of the two regimens, the cost and availability, disease status, and clinical evidence, among others (Figure 2C). Patients with high tumour burden, L858R mutation, the presence of co‐mutations like p53, CNS metastases, severe metastatic disease, younger age, good performance status, extra‐thoracic disease, and those with poor prognostic factors, including specific genetic mutations, should be considered for combination therapy in the first line, provided that they are fit to receive the combination therapy. Additionally, the AE profile of the combination regimen needs to be considered. Given the current evidence, the participating experts expressed a preference for the osimertinib plus chemotherapy regimen over the lazertinib plus amivantamab regimen, which involves intravenous (IV) administration of amivantamab. During the meeting, 54% of participants agreed that combination regimens should be discussed with patients as potentially better treatment options (Figure 2D). Furthermore, 86% of participants believed that patients with CNS metastases would benefit from the osimertinib plus chemotherapy regimen, whereas 38% recognized benefits from the amivantamab plus lazertinib regimen in this population. Moreover, 86% of participants highlighted cost and safety as the most significant differentiating factors between the osimertinib plus chemotherapy regimen and the amivantamab plus lazertinib regimen (Figure 2C,E). Further, 86% of participants prefer to treat patients with CNS metastases with osimertinib+chemotherapy regimen while 85% of the participants prefer treating young patients and those with good performance status with amivantamab+lazertinib (Figure 2E).

The drugs in the Osimertinib+chemotherapy regimen (Osimertinib, pemetrexed, cisplatin/carboplatin) have well‐established safety and efficacy profiles as compared with lazertinib plus amivantamab,^13^, ^29^ which makes the osimertinib+chemotherapy regimen more acceptable to the panel members. Additionally, the lazertinib plus amivantamab regimen is not yet available in all countries across Asia. This limitation further influences the decision‐making process regarding treatment options, as accessibility is a crucial factor in selecting the most suitable regime for patients in the region. The current status of approval and availability of drugs in both regimens is summarized in Table 2.

**TABLE 2:** Current status of approval/reimbursement in metastatic NSCLC in Asian countries (as of 28th October 2024)

Country	Regulatory approval/Reimbursement status					
	Osimertinib in first line EGFR (+) metastatic NSCLC	Osimertinib in second line EGFR T790M (+) metastatic NSCLC	Osimertinib plus Chemotherapy in first line EGFR (+) metastatic NSCLC	Lazertinib in first line EGFR (+) metastatic NSCLC	Amivantamab in EGFR Exon 20 insertion (+) metastatic NSCLC	Lazertinib + Amivantamab in first line EGFR (+) metastatic NSCLC
Australia	Yes/Yes	Yes/Yes	No/No	No/No	No/No	No/No
Bangladesh	No/No	No/No	No/No	No/No	No/No	No/No
China	Yes/Yes	Yes/Yes	Yes/No	No/No	No/No	No/No
India	Yes/No (PAP only)	Yes/No	No/No	No/No	Yes/No (PAP only)	No/No
Indonesia	Yes/No (PAP only)	Yes/PAP only	No/No	No/No	No/No	No/No
Japan	Yes/Yes	Yes/Yes	Approved	No/No	Yes/No	No/No
Malaysia	Yes/No (PAP only)	Yes/Yes (<30 patients per year)	No/No	No/No	No/No	No/No
Philippines	Yes/No (PAP only)	Yes/No (PAP only)	No/No	No/No	Yes/No	No/No
Singapore	Yes/Yes (partial reimbursement)	Yes/Yes	Yes/No	No/No	Yes/No	No/No
South Korea	Yes/Yes	Yes/Yes	Yes/No	Yes/Yes	Yes/No	No/No
Taiwan	Yes/Yes	Yes/Yes	Yes/No	No/No	Yes/No	No/No
Thailand	Yes/Yes (only denovo T790M in CSMBS)	Yes/Yes	No/No	No/No	Yes/No	No/No
Vietnam	Yes/No (PAP only)	Yes/No (PAP only)	No/No	No/No	No/No	No/No

Abbreviations: CSMBS, Civil Servant Medical‐Benefit Scheme; EGFR, Epidermal growth factor receptor; No/No, The indication has not been approved in this country nor covered by insurance; PAP, Patient assistance program; Yes/Yes, The indication has been approved in this country and is also covered by insurance.

Certain factors, such as the increased incidence of venous thromboembolism (VTE), the resultant lifelong anticoagulant administration, and injection site reactions, pose barriers to the adoption of the lazertinib plus amivantamab regimen. According to the expert panel, this regimen involves more co‐medications, which increase the probability of AEs. The experts also suggested that recommendations should be revisited once the subcutaneous formulation of amivantamab becomes available in Asia, as it is associated with an improved safety profile.^30^

The expert panel agreed that, based on efficacy, safety, accessibility, convenience of use, and current guidelines, osimertinib plus chemotherapy is the preferred treatment compared to lazertinib plus amivantamab, with 86% of participants in agreement (Figure 2F).

#### 3.1.3 What needs to be considered in managing the safety of osimertinib plus chemotherapy regimen?

*Statement*: The osimertinib plus chemotherapy regimen is acceptable for the treatment of EGFRm NSCLC patients due to its established safety profile. Dose adjustment and careful patient selection are recommended to optimize safety outcomes for this regime, based on the country's regulatory approval.

**FIGURE 3:**
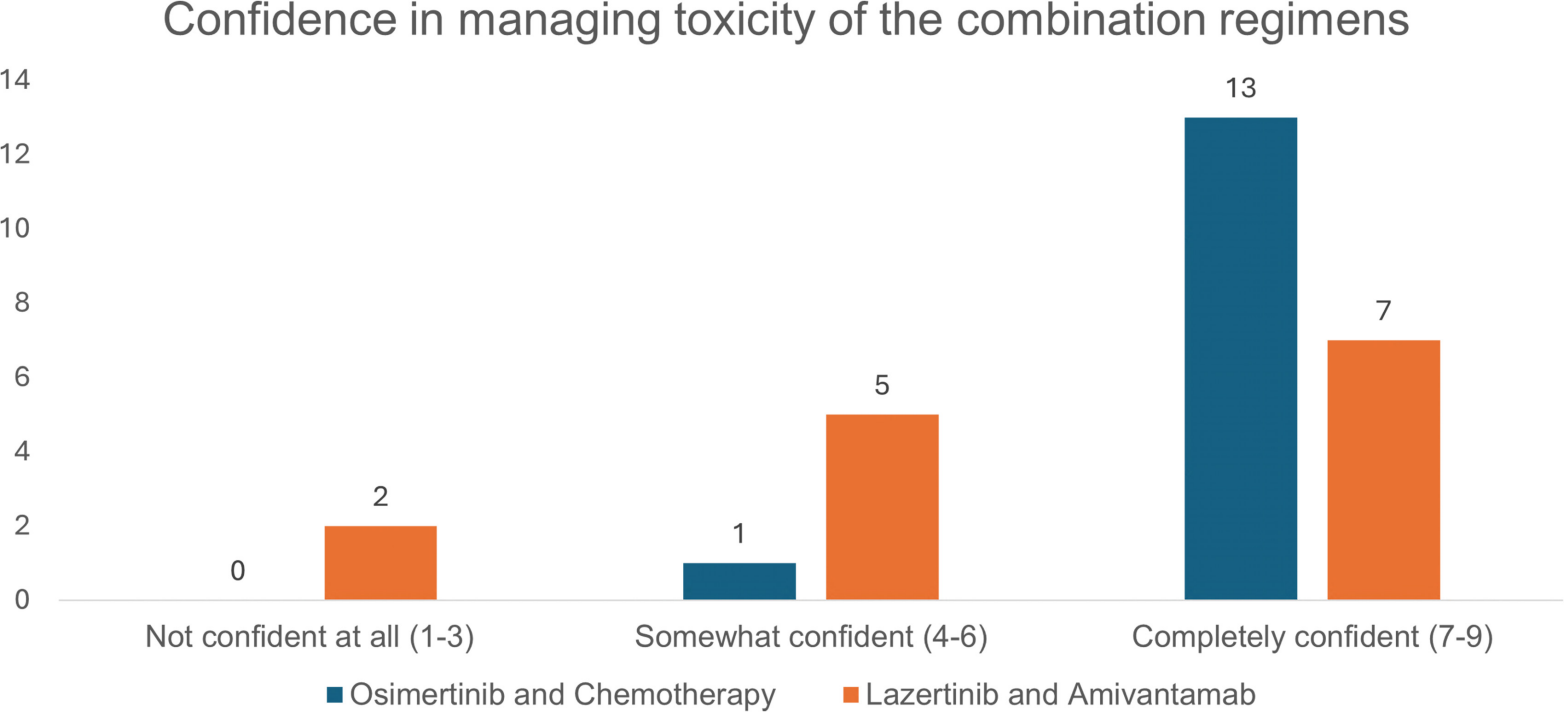
Pre‐meeting survey: Confidence in management of toxicities.

All the drugs in the osimertinib plus chemotherapy regimen are extensively used and have well‐established safety profiles. Although the combination regimen was associated with a higher incidence of grade 3 and higher AEs compared to osimertinib alone, most of the AEs reported in the trial were attributed to chemotherapy, as they appeared with the initiation of chemotherapy and subsided subsequently.^13^, ^31^ The expert panel expressed no concerns regarding the management of these AEs, given their experience with handling chemotherapy‐related toxicities. In the pre‐meeting survey, 13 out of 14 experts agreed that they were completely confident in managing the toxicity of the regimen, while the remaining participant indicated they were somewhat confident (Figure 3).

The main AE of concern for the osimertinib plus chemotherapy combination is thrombocytopenia. In the FLAURA 2 trial, 18% of patients receiving the combination regimen reported any grade of thrombocytopenia.^13^ To mitigate AEs, the experts recommend a dose reduction of osimertinib during the initial weeks of treatment, which can then be increased to the recommended dose. Another strategy suggested is providing a break from treatment after every eight cycles of combination therapy to mitigate AEs.

Additionally, patients often show reluctance to receive regimens containing chemotherapy due to safety concerns. To mitigate this reluctance, patient education is recommended. Educating patients about the treatment and its management can help alleviate concerns and improve treatment adherence.

#### 3.1.4 What needs to be considered in managing the safety of lazertinib plus amivantamab?

*Statement*: The lazertinib plus amivantamab regimen is not consistently approved and available across Asian countries. The safety profile of amivantamab presents specific concerns including infusion reactions and VTE. Measures to mitigate AEs such as the use of pre‐medication with steroids, prophylactic anticoagulants, and dose modification are recommended. Experts recommend revisiting the regimen once a subcutaneous (SC) amivantamab formulation is made available.

The combination treatment, including lazertinib plus amivantamab, is not yet available across all Asian countries. The current availability and reimbursement status of the drugs in both the osimertinib plus chemotherapy regimen and the lazertinib plus amivantamab regimen are summarized in Table 2. In the pre‐meeting survey, seven out of 14 experts expressed complete confidence in managing the toxicity of the regimen, while five indicated they were somewhat confident, and two participants were not confident in managing the toxicity of the lazertinib plus amivantamab regimen (Figure 3).

The AEs associated with the lazertinib plus amivantamab regimen are primarily attributed to EGFR and MET targeting and include paronychia, rash, diarrhoea, stomatitis, hypoalbuminemia, and peripheral oedema, as opposed to the chemotherapy‐related AEs observed with the osimertinib plus chemotherapy regimen. In trials, a higher incidence of VTEs was reported (*n* = 157/421; 37%), mostly grades 1 and 2.^29^ The use of anticoagulants is recommended for managing VTEs, along with dose reduction where necessary. Additionally, there is concern about an increased incidence of infusion‐related reactions. Due to the longer half‐life of the drugs in this regimen, adverse reactions may take longer to resolve.

SC amivantamab is associated with a lower incidence of VTEs and infusion‐related reactions compared to intravenous (IV) administration.^30^ It is recommended to revisit the safety profile of the lazertinib plus amivantamab regimen once SC amivantamab becomes available in Asian countries.

### 3.2 Optimizing therapy in the second line

**FIGURE 4:**
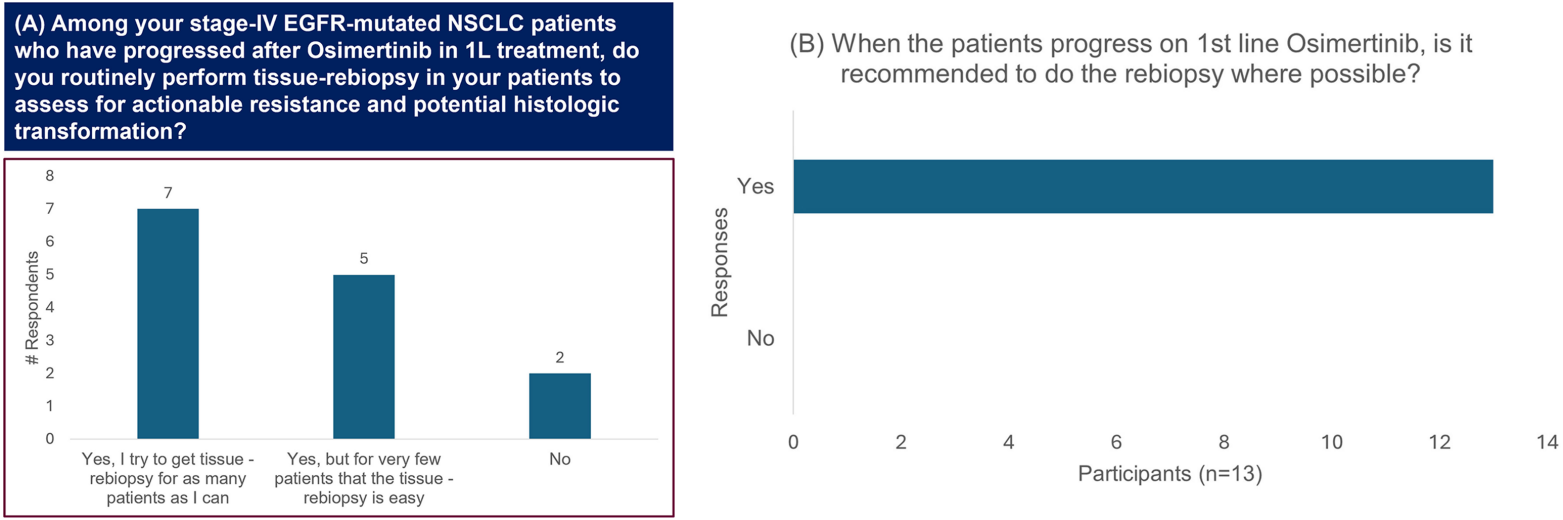
Opinion on tissue rebiopsy post progression on first‐line osimertinib. (A) pre‐meeting survey. (B) voting. EGFR, Epidermal growth factor receptor; NGS, Next generation sequencing; NSCLC, Non‐small cell lung cancer; 1L, First‐line treatment; 1st line, First‐line treatment.

In the pre‐meeting survey, 50% of participants indicated that they attempt to obtain rebiopsies for as many patients as possible, while 36% mentioned that they prefer tissue rebiopsy, though it is often difficult to perform (Figure 4A). The expert panel unanimously recommended performing a rebiopsy when a patient experiences progression on first‐line osimertinib (Figure 4B).

#### 3.2.1 What are the treatment options when the patients progress on osimertinib and chemotherapy?

*Statement*: Experts recommend continued treatment with chemotherapy (docetaxel, paclitaxel and pemetrexed) +/− immunotherapy or bevacizumab for non‐squamous NSCLC drugs or enrollment of patients in clinical trials.

Once a patient progresses on osimertinib, it is crucial to understand the type of mutation that led to resistance to plan subsequent treatment effectively. Therefore, repeat NGS testing is recommended for subsequent treatment planning. Additionally, a repeat biopsy is advised to determine whether there has been a histologic transformation of NSCLC to small cell cancer or squamous NSCLC. NGS testing on both tissue and blood is advised by the experts; however, cost constraints might limit the use of simultaneous utilization of both technologies. Besides, since rebiopsy is feasible in a limited number of patients at progression, blood‐based NGS might be utilized in such patients.

Broad panel NGS is a standard test for advanced lung adenocarcinoma, and it is strongly recommended as per the availability and local guidelines. Various co‐mutations such as p53 and CTNNB1 predict poorer prognosis, and combination treatment is suggested in such subgroups. Such personalization of treatment is possible only if broad panel NGS is performed. Hence, it is highly advisable to include a broad panel NGS in this patient population.

For patients progressing on osimertinib, treatment options are limited due to the scarcity of actionable resistance mechanisms, often restricting treatment to platinum‐based chemotherapy. The experts on the panel noted that chemotherapy might be rechallenged in patients who experience progression on rechallenging chemotherapy if recurrence occurs 6 months after discontinuing chemotherapy, with 64% of experts agreeing with this approach (as detailed in the supplementary material). Besides, another option includes the MARIPOSA‐2 regimen with amivantamab plus chemotherapy, where amivantamab is available. This regimen has shown improvement in overall response rates from 36% with chemotherapy alone to 64% with the combination.^19^

Besides, multiple ADCs targeting other molecules, such as TROP2 and HER3, are under development.^21^, ^32^ These therapies are not yet approved or available in Asia. It is recommended to enroll patients experiencing progression into clinical trials assessing these ADCs. However, a limitation exists as many ongoing trials exclude patients who have previously received chemotherapy, thereby further limiting treatment options.

Due to the limited options available after progression in most of the settings, docetaxel chemotherapy is currently recommended in Asia.

#### 3.2.2 What are the treatment options when the patients progress on lazertinib plus amivantamab regimen?

*Statement*: Experts recommend repeat NGS testing and continued treatment with chemotherapy.

MET amplification is the most common resistance mutation encountered post‐progression from osimertinib.^17^ Since amivantamab also targets MET, understanding the resistance patterns following progression on amivantamab is crucial, although these patterns are not yet fully known. Therefore, experts recommend further testing to better understand these resistance patterns and to plan subsequent treatments accordingly.

In the absence of sufficient evidence to guide treatment after progression on amivantamab, the experts recommend chemotherapy with agents such as carboplatin and pemetrexed for these patients. This approach is seen as a viable option given the current limitations in targeted therapies for resistance post‐amivantamab progression.

#### 3.2.3 In the future when the MET inhibitor + Osimertinib is available, how can we apply MET testing widely?

*Statement*: NGS testing is recommended. MET testing is considered useful; however, due to high cost and low access, the uptake is expected to be low.

Although MET amplification is the most common mechanism of resistance, NGS testing is recommended over MET‐specific testing because non‐MET mutations/amplification, such as EGFR C797S mutations, phosphatidylinositol‐4,5‐bisphosphate 3‐kinase catalytic subunit alpha (PIK3CA) mutations, ALK fusions, Neurotrophic tyrosine receptor kinase (NTRK) mutations, or combinations of these, can also be involved.^17^ MET testing is not considered mandatory for every patient due to its high cost, limited accessibility, and the requirement for additional tissue samples. This approach supports a broader understanding of resistance mechanisms, potentially identifying actionable targets beyond MET amplification.

### 3.3 Way forward and evidence gaps

The presence of circulating tumour DNA (ctDNA) is a prognostic factor where detectable ctDNA is associated with a negative prognosis and has been demonstrated in clinical trials such as the FLAURA2 and MARIPOSA trials. ctDNA presence can be correlated with the radiological response as well as clinical response.^33^, ^34^, ^35^ Recent data demonstrate that ctDNA may be applied as a molecular biomarker to predict residual disease in solid tumours. Further, the risk of relapse for early‐stage tumour is low if ctDNA is undetectable post‐treatment with curative‐intent therapy.^33^, ^34^, ^36^, ^37^ A recent non‐randomized study also demonstrated that ctDNA levels may also be used as a predictor for response while de‐escalating treatment in patients with advanced NSCLC.^38^ In the FLAURA2 and MARIPOSA trials, patients without baseline detectable ctDNA had improved progression‐free survival, as compared with those with detectable ctDNA at the baseline. However, the difference in effect was not found to be statistically significant.

ctDNA may be useful in patients in whom the primary tumour site is not accessible nor feasible to perform a biopsy. Additionally, ctDNA may guide treatment selection in patients with mixed responses and provide insights on the mechanism of resistance. Testing using a complementary approach with tissue biopsy as well as liquid biopsy may be associated with a higher yield and pick up rate of mutations.

Certain experts in the panel agreed that there is a necessity to incorporate ctDNA in the diagnosis; however, other experts suggested the role of ctDNA to be more research‐oriented, rather than for clinical use. Further, ctDNA testing is not reimbursed in most Asian countries, limiting the uptake of testing. Hence, further research and deliberation are necessary to determine the role of ctDNA in the diagnosis and management of NSCLC.

Certain gaps in evidence were identified. Since the overall survival data from the trials is not yet mature, it is difficult to determine which of the two regimens between osimertinib plus chemotherapy and lazertinib plus amivantamab is superior in the long term. Long‐term follow‐up data will be required to reach a definitive conclusion. Additionally, neither trial includes patients with uncommon EGFR mutations, so there is no available evidence on how these patients respond to these regimens. A clinical trial addressing these outcomes is highly desirable.

Considering the safety implications of the combination regimens, it would be beneficial to assess the impact of a limited number of chemotherapy cycles on safety and efficacy outcomes in this patient population. Moreover, the role of continuing osimertinib in patients who have already progressed on it needs further evaluation. Further data regarding acquired resistance and quality of life data for safety management is desirable. The data regarding potential resistance patterns for amivantamab is evolving, and more evidence would aid in making appropriate treatment selections. Comprehensive genetic profiling of patients post‐progression may help make second‐line therapy more tailored. Furthermore, there is a lack of evidence on the clinical outcomes with ADC treatments.

### 3.4 Limitations

The expert opinions expressed in this study should be interpreted rationally as these are the opinions of a limited group of experts, and other expert panels may not reach the same conclusions. Furthermore, the opinions expressed in this study have temporal validity, as they may change over time, and participant views were not unanimous on all topics.

## 4 CONCLUSION

Lung cancer experts from Asia recommend NGS for the appropriate selection of patients for EGFR TKI treatment. The experts emphasize selecting patients with specific clinical profiles for the combination regimen to balance the risk–benefit ratio. Most participants favored the osimertinib plus chemotherapy regimen over lazertinib plus amivantamab due to its familiarity with AE management, cost, and availability in the Asia region. Additionally, recommendations were made regarding the management of safety profiles for both regimens and the management of NSCLC patients post‐progression on osimertinib.

## AUTHOR CONTRIBUTIONS

**Gee‐Chen Chang:** Conceptualization; funding acquisition; writing – original draft; writing – review and editing; visualization; validation; methodology; data curation; supervision; project administration; formal analysis. **Akhil Kapoor:** Conceptualization; data curation; formal analysis; writing – original draft; writing – review and editing; validation; visualization. **Chee Khoon Lee:** Conceptualization; writing – original draft; writing – review and editing; visualization; validation; formal analysis; data curation. **Chunxia Su:** Conceptualization; writing – original draft; writing – review and editing; validation; visualization; formal analysis; data curation. **Daniel Chan:** Conceptualization; writing – original draft; validation; visualization; writing – review and editing; formal analysis; data curation. **Guia Elena Imelda Ladrera:** Conceptualization; writing – original draft; writing – review and editing; visualization; validation; formal analysis; data curation. **Hye Ryun Kim:** Conceptualization; writing – original draft; validation; visualization; writing – review and editing; formal analysis; data curation. **Mostafa Aziz Sumon:** Conceptualization; writing – original draft; validation; visualization; writing – review and editing; data curation; formal analysis. **Moushumi Suryavanshi:** Conceptualization; writing – original draft; validation; visualization; writing – review and editing; formal analysis; data curation. **Sita Andarini:** Conceptualization; writing – original draft; validation; formal analysis; data curation; visualization; writing – review and editing. **Tatsuya Yoshida:** Conceptualization; writing – original draft; validation; visualization; writing – review and editing; formal analysis; data curation. **Thanyanan Reungwetwattana:** Conceptualization; writing – original draft; validation; visualization; writing – review and editing; formal analysis; data curation. **Tuan Khoi Nguyen:** Conceptualization; writing – original draft; validation; visualization; writing – review and editing; formal analysis; data curation. **Pei Jye Voon:** Conceptualization; writing – original draft; validation; visualization; writing – review and editing; formal analysis; data curation.

## FUNDING INFORMATION

The study, meetings, literature review, medical writing, and publication of this manuscript were funded by AstraZeneca.

## CONFLICT OF INTEREST STATEMENT

Gee‐Chen Chang has received honoraria from AstraZeneca, Merck Sharp and Dohme, Novartis, Boehringer Ingelheim, Hoffman‐La Roche, Eli Lilly, Pfizer, and Bristol‐Myers Squibb. Akhil Kapoor has served as an investigator on multiple clinical trials for AstraZeneca, Bayer, Bristol Myers Squibb, Eli Lilly, Erixis, MSD, and Novartis. All grants in this regard were paid to the institution. Chee Khoon Lee has received honoraria and grants from AstraZeneca, Amgen, Janssen, GSK, Boehringer Ingelheim, MSD, Roche, Gilead, Novartis, and Glenmark, and meeting/travel support from AstraZeneca and Janssen. Chunxia Su declares no conflict of interest for this study. Daniel Chan and Moushumi Suryavanshi have received consulting fees from AstraZeneca. Guia Elena Imelda Ladrera has served on the advisory board and received honoraria and travel expenses from AstraZeneca. Hye Ryun Kim received honoraria from AstraZeneca, Bristol Myers Squibb, Genentech/Roche, stock ownership in Bridgebio Therapeutics; served in a consultation or advisory role for Bayer, AstraZeneca, Bristol Myers Squibb, Takeda, and Yuhan; and received research funding from the Yonsei Lee Youn Jae Fellowship outside of the current study. Mostafa Aziz Sumon reports no conflict of interest. Sita Andarini has received honoraria from AstraZeneca, Darya Varia, Etana, GOF, Hetero, JNJ, Kalgen Innolab, MSD, Pfizer, Roche, Takeda, ZuelligPharma Therapeutics, travel grants from AstraZeneca, Darya Varia, Etana, GOF, Hetero, JNJ, Kalgen Innolab, MSD, Pfizer, Roche, Takeda, ZuelligPharma Therapeutics, and has served on advisory boards for AstraZeneca, Darya Varia, JNJ, MSD. Tatsuya Yoshida has received grants from Novartis, AbbVie, Amgen, Daiichi‐Sankyo, AstraZeneca, MSD, Chugai Pharmaceutical Co. Ltd, Astellas, Boehringer Ingelheim, BMS, Ono Pharmaceutical, and Merck Biopharma, honoraria from Novartis, Daiichi‐Sankyo, AstraZeneca, MSD, Chugai Pharmaceutical Co. Ltd, BMS, Ono, Takeda, Pfizer, Lilly, and Merck Biopharma, and has been a member of the advisory board for Novartis, MSD, Amgen, Chugai Pharmaceutical Co. Ltd, Pfizer, and Boehringer Ingelheim. Thanyanan Reungwetwattana received honoraria from AstraZeneca, Roche, BMS, J&J, Pfizer, Amgen, Takeda, MSD; served on advisory board for AstraZeneca, Roche, BMS, J&J, Pfizer, Amgen, Takeda, Yuhan, MSD; and a grant for clinical research was paid to the institution by AstraZeneca, Roche, MSD, Yuhan. Tuan Khoi Nguyen has received grants from MSD, Roche, and AstraZeneca; honoraria, travel support, and consulting fees from MSD, Roche, AstraZeneca, Novartis, Pfizer, and Pierre Fabre. Pei Jye Voon has received grants/contracts from Astra Zeneca, Novartis, Boehringer Ingelheim, Janssen‐Cilag, Johnson & Johnson, Viracta Therapeutics Inc., ROCHE, Merck KGaA, Merck Sharp & Dohme, Beigene, Amgen, Revolutionary Medicine; consulting fees from Astra Zeneca, Novartis, Merck Sharp & Dohme, Pfizer, Beigene, Amgen, Merck KGaA, Janssen‐Cilag, Johnson & Johnson; and honoraria payments from Astra Zeneca, Novartis, Merck Sharp & Dohme, Pfizer, Amgen, Merck KGaA, Janssen‐Cilag, Johnson & Johnson.

## Supporting information


**DATA S1.** Supporting Information.

## Data Availability

All data generated or analyzed during this study are included in this published article/as supplementary information files. Further information is available from the corresponding author upon request.

## References

[1] Cancer Today [Internet]. https://gco.iarc.who.int/today/

[2] Zheng M . Classification and pathology of lung cancer. Surg Oncol Clin N Am. 2016;25(3):447‐468. 27261908 10.1016/j.soc.2016.02.003

[3] Lam WK . Lung cancer in Asian women‐the environment and genes. Respirology. 2005;10(4):408‐417. 16135162 10.1111/j.1440-1843.2005.00723.x

[4] Lam DC , Liam CK , Andarini S , et al. Lung cancer screening in Asia: an expert consensus report. J Thorac Oncol. 2023;18:1303‐1322. 37390982 10.1016/j.jtho.2023.06.014

[5] Zhou W , Christiani DC . East meets West: ethnic differences in epidemiology and clinical behaviors of lung cancer between east Asians and Caucasians. Chin J Cancer. 2011;30(5):287‐292. 21527061 10.5732/cjc.011.10106 PMC4013393

[6] Mitsudomi T , Yatabe Y . Epidermal growth factor receptor in relation to tumor development: EGFR gene and cancer. FEBS J. 2010;277(2):301‐308. 19922469 10.1111/j.1742-4658.2009.07448.x

[7] Sacher AG , Paweletz C , Dahlberg SE , et al. Prospective validation of rapid plasma genotyping as a sensitive and specific tool for guiding lung cancer care. JAMA Oncol. 2016;2(8):1014‐1022. 27055085 10.1001/jamaoncol.2016.0173 PMC4982795

[8] Wu YL , Planchard D , Lu S , et al. Pan‐Asian adapted clinical practice guidelines for the management of patients with metastatic non‐small‐cell lung cancer: a CSCO‐ESMO initiative endorsed by JSMO, KSMO, MOS, SSO and TOS. Ann Oncol Off J Eur Soc Med Oncol. 2019;30(2):171‐210. 10.1093/annonc/mdy554 30596843

[9] Lin JJ , Cardarella S , Lydon CA , et al. Five‐year survival in EGFR‐mutant metastatic lung adenocarcinoma treated with EGFR‐TKIs. J Thorac Oncol. 2016;11(4):556‐565. 26724471 10.1016/j.jtho.2015.12.103 PMC4979601

[10] Shimamura SS , Shukuya T , Asao T , et al. Survival past five years with advanced, EGFR‐mutated or ALK‐rearranged non‐small cell lung cancer‐is there a “tail plateau” in the survival curve of these patients? BMC Cancer. 2022;22(1):323. 35337281 10.1186/s12885-022-09421-7 PMC8953392

[11] Ramalingam SS , Vansteenkiste J , Planchard D , et al. Overall survival with Osimertinib in untreated, EGFR‐mutated advanced NSCLC. N Engl J Med. 2020;382(1):41‐50. 31751012 10.1056/NEJMoa1913662

[12] NCCN . NCCN Clinical Practice Guidelines in Oncology (NCCN Guidelines®) for Management of non‐small Cell Lung Cancer [Internet]. NCCN. 2024 https://www.nccn.org/professionals/physician_gls/pdf/nscl.pdf

[13] Planchard D , Jänne PA , Cheng Y , et al. Osimertinib with or without chemotherapy in EGFR‐mutated advanced NSCLC. N Engl J Med. 2023;389(21):1935‐1948. 37937763 10.1056/NEJMoa2306434

[14] Cho BC , Kim DW , Spira AI , et al. Amivantamab plus lazertinib in osimertinib‐relapsed EGFR‐mutant advanced non‐small cell lung cancer: a phase 1 trial. Nat Med. 2023;29(10):2577‐2585. 37710001 10.1038/s41591-023-02554-7 PMC10579096

[15] Cho BC , Han J , Kim S , et al. MA26.09 Lazertinib, a third generation EGFR‐TKI, in patients with EGFR‐TKI‐resistant NSCLC: updated results of a phase I/II study. J Thorac Oncol. 2018;13(10):S453.

[16] Cho BC , Felip E , Hayashi H , et al. MARIPOSA: phase 3 study of first‐line amivantamab + lazertinib versus osimertinib in EGFR‐mutant non‐small‐cell lung cancer. Future Oncol. 2022;18(6):639‐647. 34911336 10.2217/fon-2021-0923

[17] Lee CK , Robichaux JP , Jänne PA , et al. 514MO acquired mechanisms of resistance to first‐line (1L) osimertinib with or without platinum‐based chemotherapy (CT) in EGFR‐mutated (EGFRm) advanced NSCLC: preliminary data from FLAURA2. Ann Oncol. 2023;34:S1669‐S1670.

[18] Piper Vallillo AJ , Viray H , Feldman J , Rangachari D . Management of Treatment Resistance in patients with advanced epidermal growth factor receptor–mutated lung cancer: personalization, parsimony, and partnership. J Clin Oncol. 2024;42(11):1215‐1221. 38412397 10.1200/JCO.23.02417

[19] Passaro A , Wang J , Wang Y , et al. Amivantamab plus chemotherapy with and without lazertinib in EGFR‐mutant advanced NSCLC after disease progression on osimertinib: primary results from the phase III MARIPOSA‐2 study. Ann Oncol. 2024;35(1):77‐90. 37879444 10.1016/j.annonc.2023.10.117

[20] Shimizu T , Sands J , Yoh K , et al. First‐in‐human, phase I dose‐escalation and dose‐expansion study of trophoblast cell‐surface antigen 2‐directed antibody‐drug conjugate Datopotamab Deruxtecan in non‐small‐cell lung cancer: TROPION‐PanTumor01. J Clin Oncol off J Am Soc Clin Oncol. 2023;41(29):4678‐4687. 10.1200/JCO.23.00059 PMC10564307 37327461

[21] Paz‐Ares L , Ahn MJ , Lisberg AE , et al. 1314MO TROPION‐Lung05: Datopotamab deruxtecan (Dato‐DXd) in previously treated non‐small cell lung cancer (NSCLC) with actionable genomic alterations (AGAs). Ann Oncol. 2023;34:S755‐S756.

[22] Berger MF , Mardis ER . The emerging clinical relevance of genomics in cancer medicine. Nat Rev Clin Oncol. 2018;15(6):353‐365. 29599476 10.1038/s41571-018-0002-6 PMC6658089

[23] Kamps R , Brandão RD , Bosch BJ , et al. Next‐generation sequencing in oncology: genetic diagnosis, risk prediction and cancer classification. Int J Mol Sci. 2017;18(2):308. 28146134 10.3390/ijms18020308 PMC5343844

[24] Loong HH , Shimizu T , Prawira A , et al. Recommendations for the use of next‐generation sequencing in patients with metastatic cancer in the Asia‐Pacific region: a report from the APODDC working group. ESMO Open. 2023;8(4):101586. 37356359 10.1016/j.esmoop.2023.101586 PMC10319859

[25] Mehta A , Vasudevan S , Sharma SK , et al. Biomarker testing for advanced lung cancer by next‐generation sequencing; a valid method to achieve a comprehensive glimpse at mutational landscape. Appl Cancer Res. 2020;40:4.

[26] Tan DSP , Tan DSW , Tan IBH , et al. Recommendations to improve the clinical adoption of NGS‐based cancer diagnostics in Singapore. Asia Pac J Clin Oncol. 2020;16(4):222‐231. 32301274 10.1111/ajco.13339 PMC7496576

[27] Mitsudomi T , Tan D , Yang JCH , et al. Expert consensus recommendations on biomarker testing in metastatic and nonmetastatic NSCLC in Asia. J Thorac Oncol. 2023;18(4):436‐446. 36379356 10.1016/j.jtho.2022.10.021

[28] Li T , Ma W , Al‐Obeidi E . Evolving precision first‐line systemic treatment for patients with unresectable non‐small cell lung cancer. Cancer. 2024;16(13):2350. 10.3390/cancers16132350 PMC11240640 39001412

[29] Cho BC , Felip E , Spira AI , et al. LBA14 Amivantamab plus lazertinib vs osimertinib as first‐line treatment in patients with EGFR‐mutated, advanced non‐small cell lung cancer (NSCLC): primary results from MARIPOSA, a phase III, global, randomized, controlled trial. Ann Oncol. 2023;34:S1306.

[30] Leighl NB , Akamatsu H , Lim SM , et al. Subcutaneous amivantamab vs intravenous amivantamab, both in combination with lazertinib, in refractory EGFR‐mutated, advanced non‐small cell lung cancer (NSCLC): primary results, including overall survival (OS), from the global, phase 3, randomized controlled PALOMA‐3 trial. J Clin Oncol. 2024;42(17_suppl):LBA8505. 10.1200/JCO.24.01001 PMC11469630 38857463

[31] Planchard D , Jänne PA , Cheng Y , et al. LBA68 FLAURA2: safety and CNS outcomes of first‐line (1L) osimertinib (osi) ± chemotherapy (CTx) in EGFRm advanced NSCLC. Ann Oncol. 2023;34:S1311‐S1312.

[32] Yu HA , Goto Y , Hayashi H , et al. HERTHENA‐Lung01, a phase II trial of Patritumab Deruxtecan (HER3‐DXd) in epidermal growth factor receptor–mutated non–small‐cell lung cancer after epidermal growth factor receptor tyrosine kinase inhibitor therapy and platinum‐based chemotherapy. J Clin Oncol. 2023;41(35):5363‐5375. 37689979 10.1200/JCO.23.01476 PMC10713116

[33] Chaudhuri AA , Chabon JJ , Lovejoy AF , et al. Early detection of molecular residual disease in localized lung cancer by circulating tumor DNA profiling. Cancer Discov. 2017;7(12):1394‐1403. 28899864 10.1158/2159-8290.CD-17-0716 PMC5895851

[34] Moding EJ , Liu Y , Nabet BY , et al. Circulating tumor DNA dynamics predict benefit from consolidation immunotherapy in locally advanced non‐small‐cell lung cancer. Nat Cancer. 2020;1(2):176‐183. 34505064 10.1038/s43018-019-0011-0 PMC8425388

[35] Ding PN , Becker TM , Bray VJ , et al. The predictive and prognostic significance of liquid biopsy in advanced epidermal growth factor receptor‐mutated non‐small cell lung cancer: a prospective study. Lung Cancer. 2019;134:187‐193. 31319980 10.1016/j.lungcan.2019.06.021

[36] Jung HA , Ku BM , Kim YJ , et al. Longitudinal monitoring of circulating tumor DNA from plasma in patients with curative resected stages I to IIIA EGFR‐mutant non–small cell lung cancer. J Thorac Oncol. 2023;18(9):1199‐1208. 37308037 10.1016/j.jtho.2023.05.027

[37] Zhang JT , Liu SY , Gao W , et al. Longitudinal undetectable molecular residual disease defines potentially cured population in localized non‐small cell lung cancer. Cancer Discov. 2022;12(7):1690‐1701. 35543554 10.1158/2159-8290.CD-21-1486 PMC9394392

[38] Dong S , Wang Z , Zhang JT , et al. Circulating tumor DNA‐guided De‐escalation targeted therapy for advanced non−small cell lung cancer: a nonrandomized controlled trial. JAMA Oncol. 2024;10(7):932‐940. 38869865 10.1001/jamaoncol.2024.1779 PMC12312504

