# Supplementary material for: Optimizing management of stage IV EGFR mutant non‐small cell lung cancer in Asia: An expert opinion
Source: Int J Cancer. 2025 Jun 14;157(8):1648–61. doi: 10.1002/ijc.35512 (PMC12375841; doi:10.1002/ijc.35512)
Supplement: Supplementary file 1 — DATA S1. Supporting Information. [file IJC-157-1648-s001.pdf]

Optimizing management of stage IV EGFR mutant non-small cell lung cancer in Asia – An expert opinion

Short title: Expert opinion on management of EGFR mutant NSCLC in Asia

Authors: Gee-Chen Chang, Akhil Kapoor, Chee Khoon Lee, Chunxia Su, Daniel Chan, Guia Elena Imelda Ladrera, Hye Ryun Kim, Mostafa Aziz Sumon, Moushumi Suryavanshi, Sita Andarini, Tatsuya Yoshida, Thanyanan Reungwetwattana, Tuan Khoi Nguyen, Pei Jye Voon

Contents

Pre-meeting survey questions and responses 2

Voting questions and responses 15

Pre-meeting survey questions and responses

Q1: In your clinical practice, how many percent of non-squamous cell carcinoma stage IV NSCLC patients have EGFR test at the time of diagnosis?

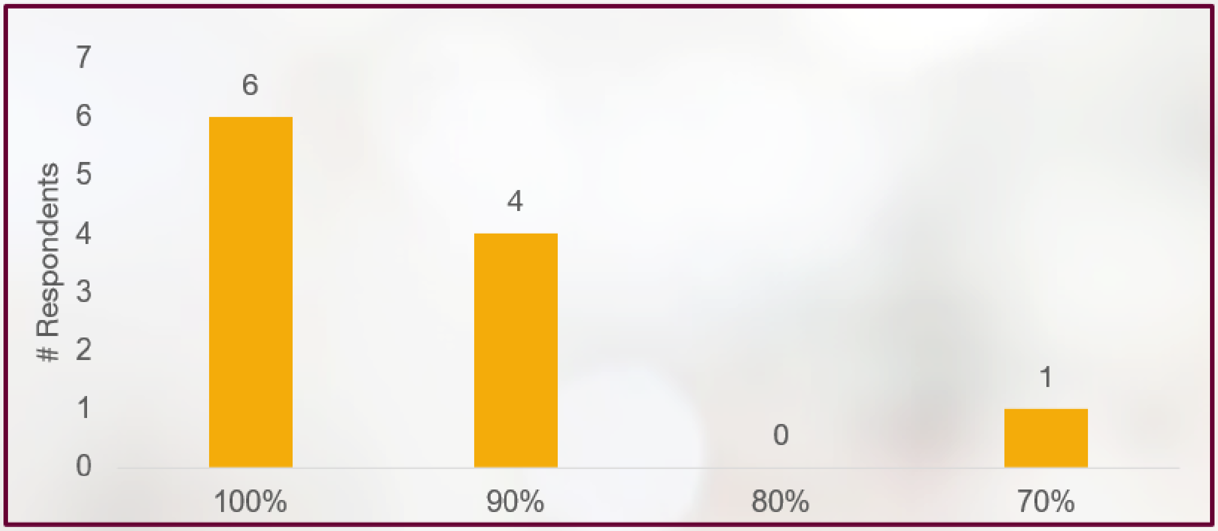

- 100% (6/11)
- 90% (4/11)
- 80% (0/11)
- 70% (1/11)

Others, please specify

- 50% (2/3)
- 60% (1/3)

Q2: For newly diagnosed stage-IV EGFR-mutated NSCLC patients (Del19 and L858R), which of the following EGFR-TKI options is the most preferred as the 1L treatment in your current practice?

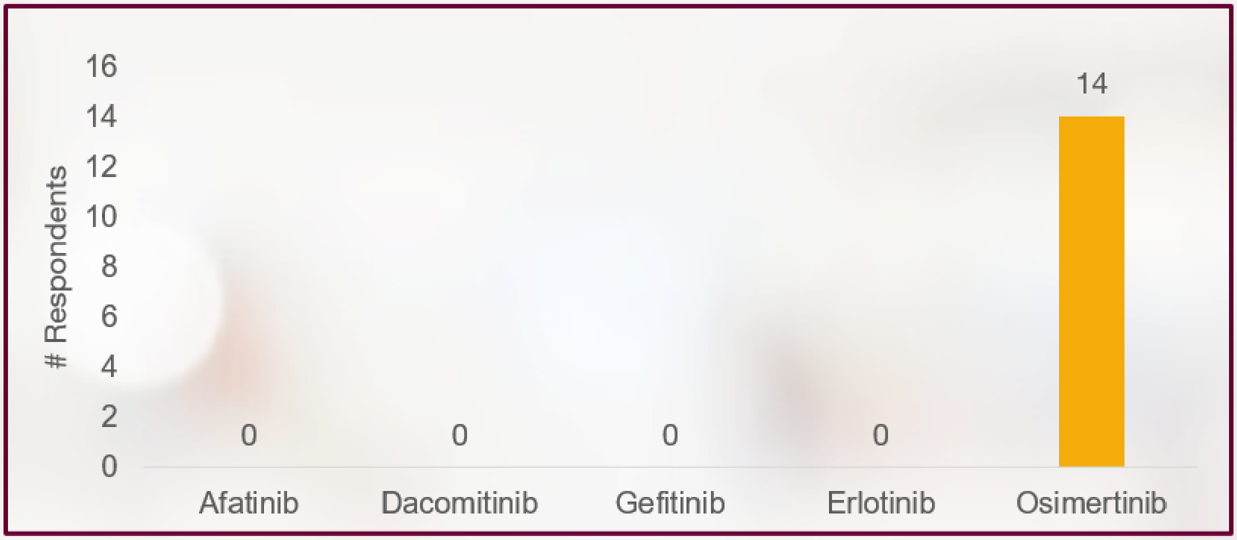

- Afatinib (0/14)
- Dacomitinib (0/14)
- Gefitinib (0/14)
- Erlotinib (0/14)
- Osimertinib (14/14)

Q3: What is your current management for patients with EGFR exon 20 insertions mutant NSCLC?

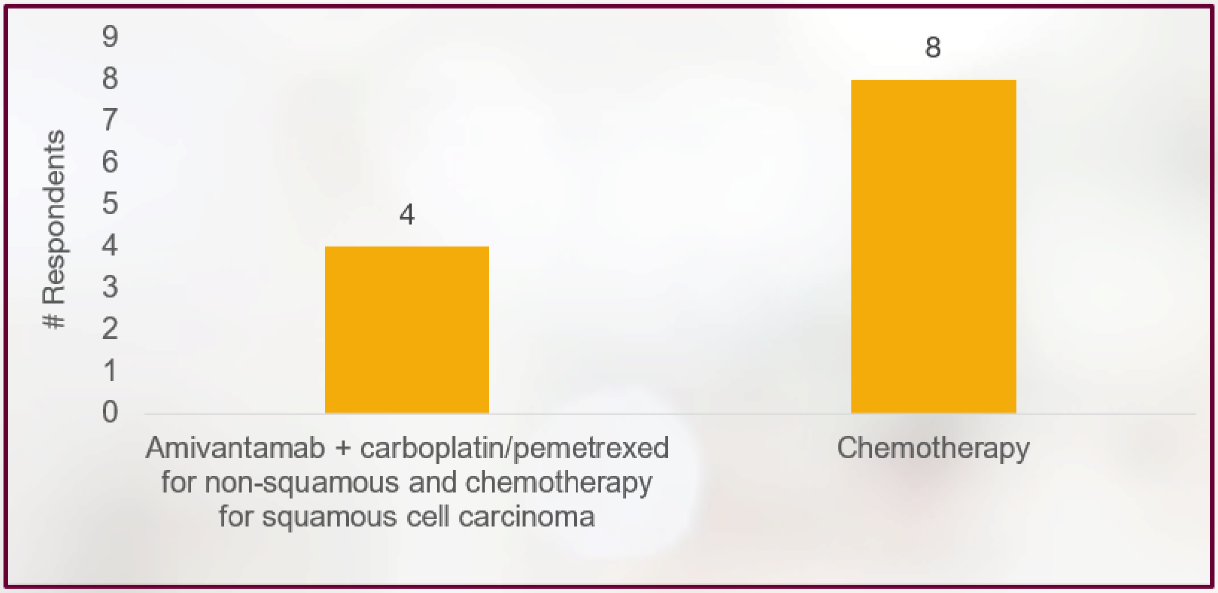

Others, please specify

Responses from 2 experts

- Try TKI, Chemotherapy or amivantamab (1/2)
- Clinical trial (1/2)

Q4: What is your current management for patients with uncommon EGFR mutation (S768I, L861Q, and/or G719X) NSCLC?

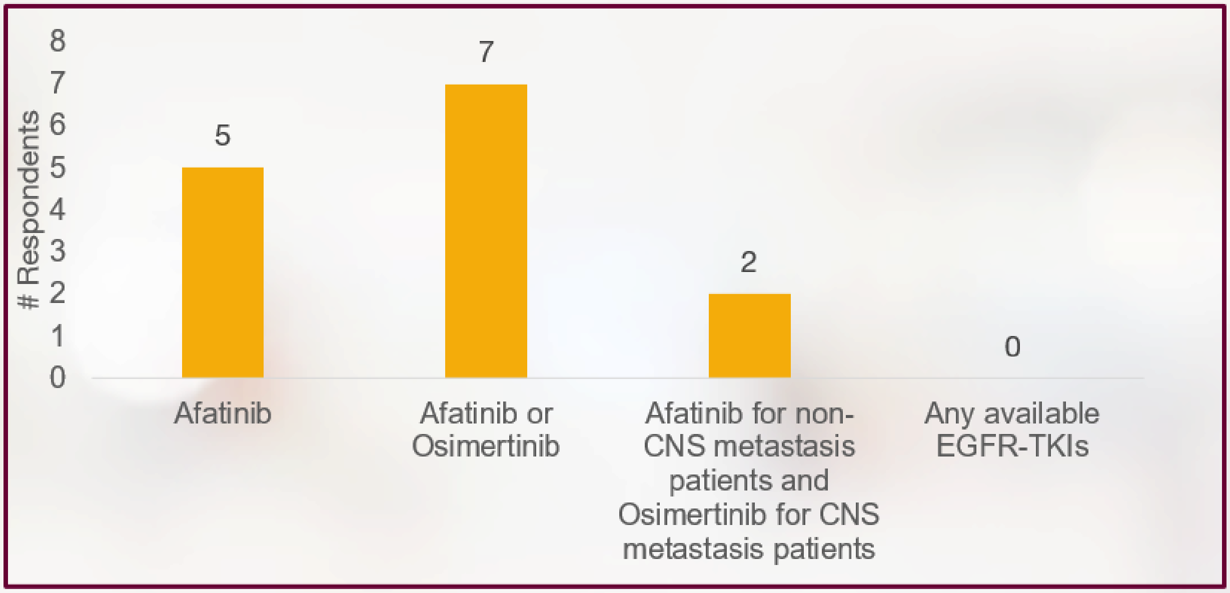

Others, please specify

- None

Q5: Based on the data from FLAURA 2, what would be the implication of combination regimen of Osimertinib and chemotherapy in your future clinical practice?

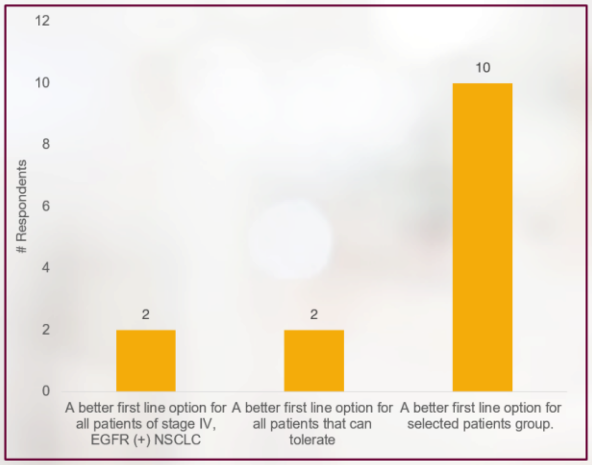

A better first line option for all patients that can tolerate. Please specify the predicative factors for tolerance.

Responses from 2 experts
- Performance status (2/2)
- Age (2/2)

A better first line option for selected patients group. Please specify the profile of patients

Responses from 10 experts
- brain metastasis/brain mets/CNS mets (7/10)
- L858R (2/10)
- high tumor burden (2/10)
- younger patient (2/10)
- fit patient with good ECOG status that can withstand chemotherapy (1/10)
- Other high risk patients with heavy disease burden (1/10)
- TP53 mutant, PACC mutations (1/10)
- pts with combination with other marker→ KRAS, P53 etc. (1/10)
- Liver mets (1/10)
- Exon 21, p53 mutant (1/10)
- Multiple meta (1/10)

Q6: Based on the data from MARIPOSA, what would be the implication of combination regimen of Amivantamab + Lazertinib in your future clinical practice?

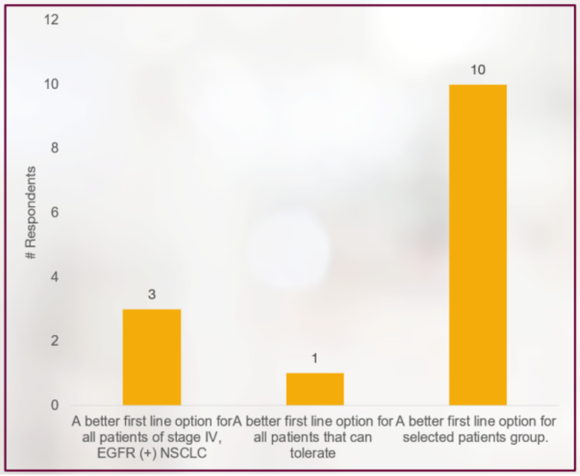

A better first line option for all patients that can tolerate. Please specify the predicative factors for tolerance.

Responses from 1 expert
- Age, performance status, cost

A better first line option for selected patients group. Please specify the profile of patients

Responses from 10 experts
- brain metastasis/ brain mets/ difficult brain mets/ CNS mets (5/10)
- Liver metastasis/liver mets (3/10)
- Young (3/10)
- high tumor burden (3/10)
- Good PS (2/10)
- Concurrent mutation (2/10)
- High risk patient (1/10)
- TP53 PACC mutations (1/10)
- refusal of chemotherapy (1/10)
- L858R (1/10)
- Met amplified (1/10)
- Poor prognostic groups (1/10)
- MET amplification at baseline together with EGFRm (1/10)

Q7: What would be the differentiation factors between the two regimens, FLAURA 2 and MARIPOSA, when you consider for future application?

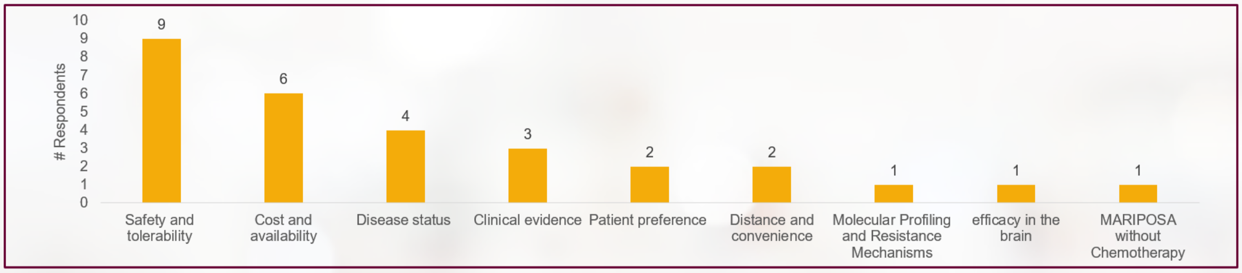

Responses from 14 experts
- Safety and tolerability (9/14)
- Cost and availability (6/14)
- Disease status (4/14)
- Clinical evidence (3/14)
- Patient preference (2/14)
- Distance and convenience (2/14)
- Molecular Profiling and Resistance Mechanisms (1/14)
- Efficacy in the brain (1/14)
- MARIPOSA without Chemotherapy (1/14)

Q8: Have you got the experience with combination regimen in FLAURA 2 or MARIPOSA? (inside or outside clinical trials)?

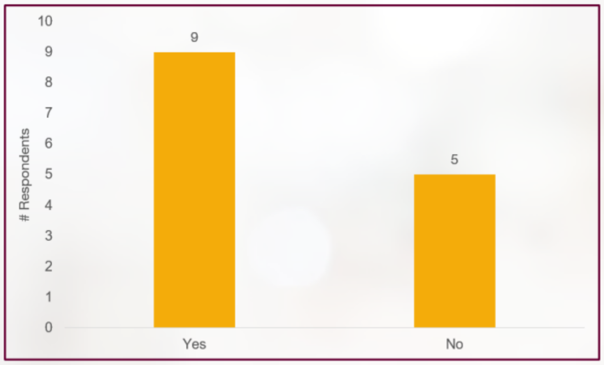

- Yes (9/14)
- No (5/14)

If 'Yes', please specify the details (outside of inside clinical trials? Which trial? Experience with patient profile, efficacy, safety?

# Expert	Responses given by experts
Expert 1	• FLAURA2→both inside or outside trials<br>• Mariposa→ inside trial
Expert 2	• MARIPOSA
Expert 3	• Inside clinical trial for amivantamab+lazertinib. Cutaneous toxicities, *we* need experience to manage; Efficacy is good.
Expert 4	• Outside trials
Expert 5	• Inside clinical trial
Expert 6	• FLAURA2; Chrysalis
Expert 7	• FLAURA2 is routinely utilized at our center in the poor prognostic group. Toxicity is definitely increased as compared to Osimertinib alone, but the efficacy is also improved proportionally.
Expert 8	• Yes
Expert 9	• Inside clinical trials

Q9: Based on the safety data from FLAURA 2, what is your confidence level in managing the toxicity of the regimen?

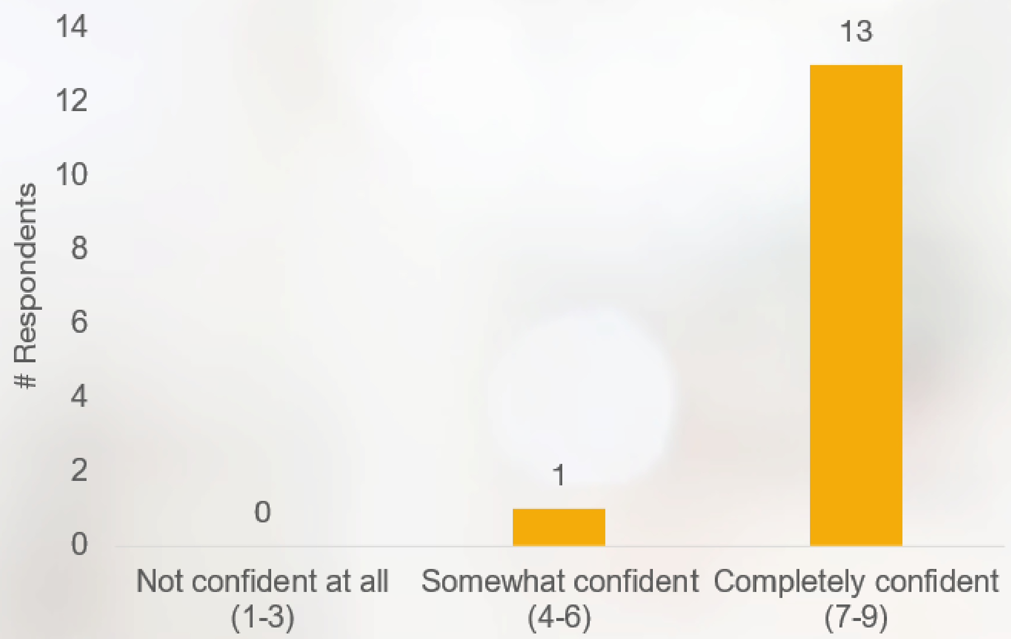

13 out of 14 respondents expressed that they are completely confident in managing the toxicity of the regimen based on the safety data from FLAURA 2

Q10: Based on the safety data from MARIPOSA, what is your confidence level in managing the toxicity of the regimen?

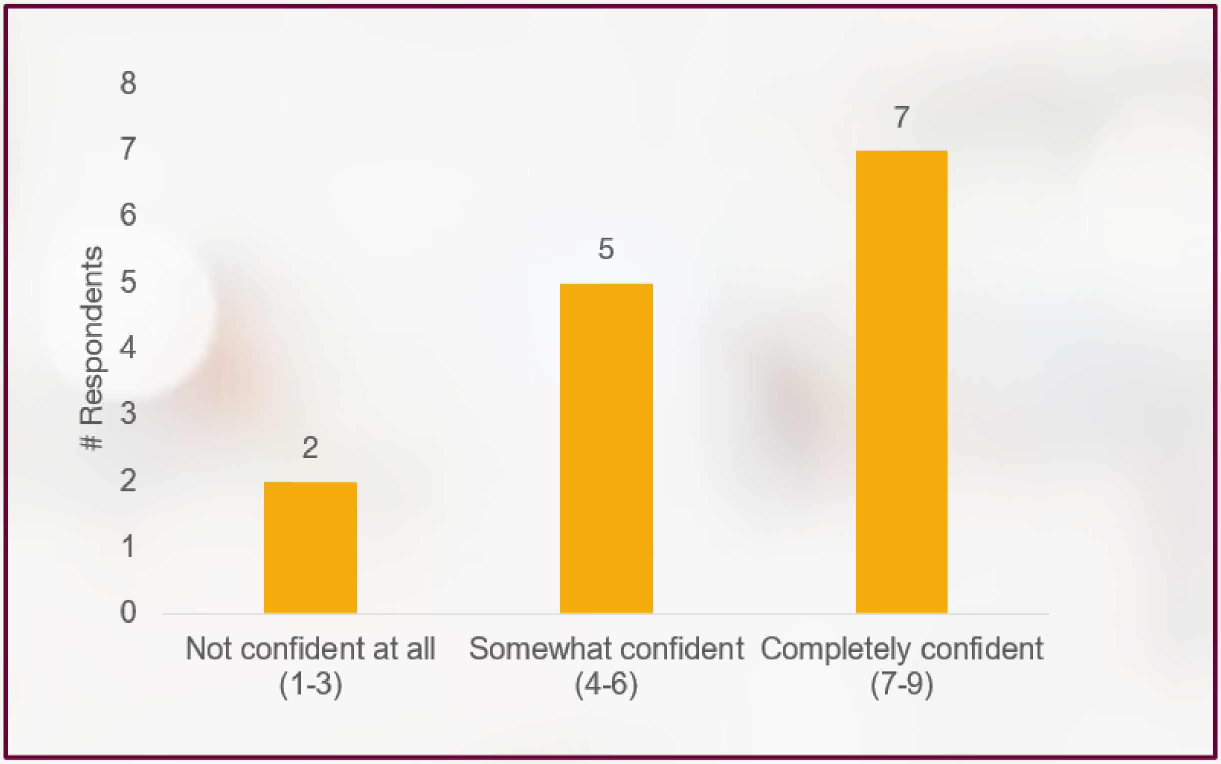

7 out of 14 respondents expressed that they are completely confident in managing the toxicity of the regimen based on the safety data from MARIPOSA

Q11: What are the important attributes when selecting 1L treatment option for stage-IV EGFR-mutated NSCLC patients? Could you help us to rate all the attributes below on a scale of 1-7, 7 means "the most important"

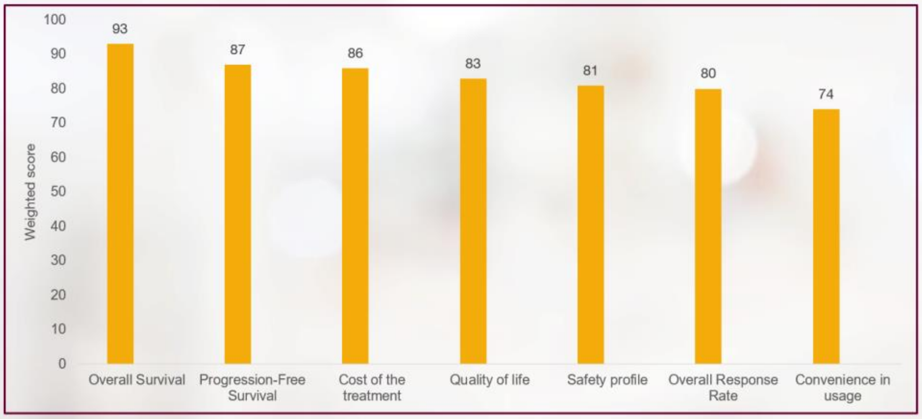

Responses from 14 experts

- Overall survival followed by Progression-Free survival and Cost of the treatment were rated as top 3 most important attributes when selecting 1L treatment option for stage-IV EGFR-mutated NSCLC patients by the respondents.

Others, please specify

Responses from 3 experts

- availability in UHC (1/3)
- Availability (1/3)
- Patient access program (1/3)

Q12: Among your stage-IV EGFR-mutated non-small-cell lung cancer (NSCLC) patients who have progressed after first and second generation of EGFR-TKIs in 1L treatment, how do you perform EGFR test for them to find T790M mutation?

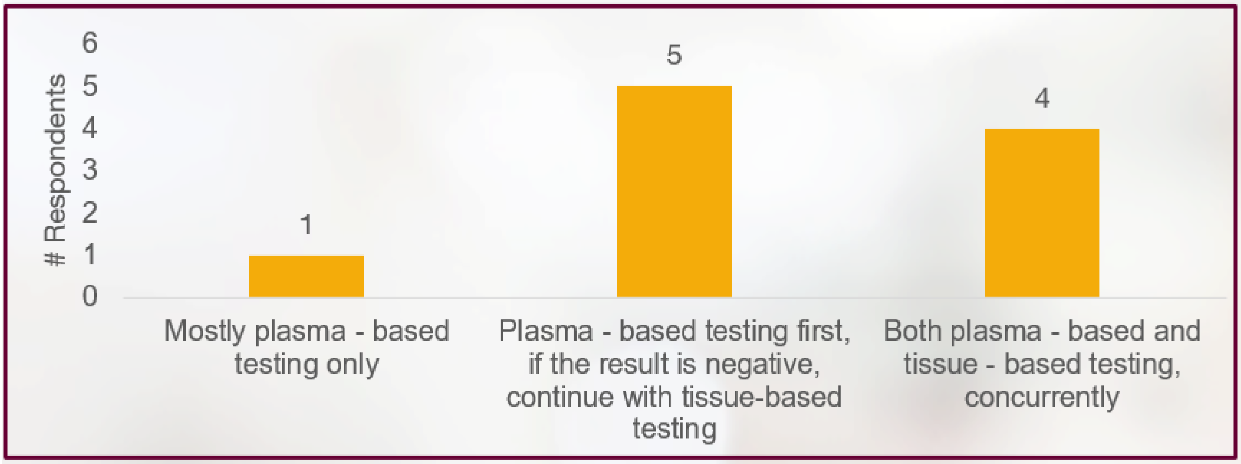

Others, please specify

Responses from 4 experts

- tissue first, for drug reimbursement (1/4)
- mostly plasma, if possible rebiopsy (1/4)
- Tissue only (1/4)
- Biopsy on progressing lesion (1/4)

Q13: Among your stage-IV EGFR-mutated NSCLC patients who have progressed after Osimertinib in 1L treatment, do you routinely perform tissue-rebiopsy in your patients to assess for actionable resistance and potential histologic transformation?

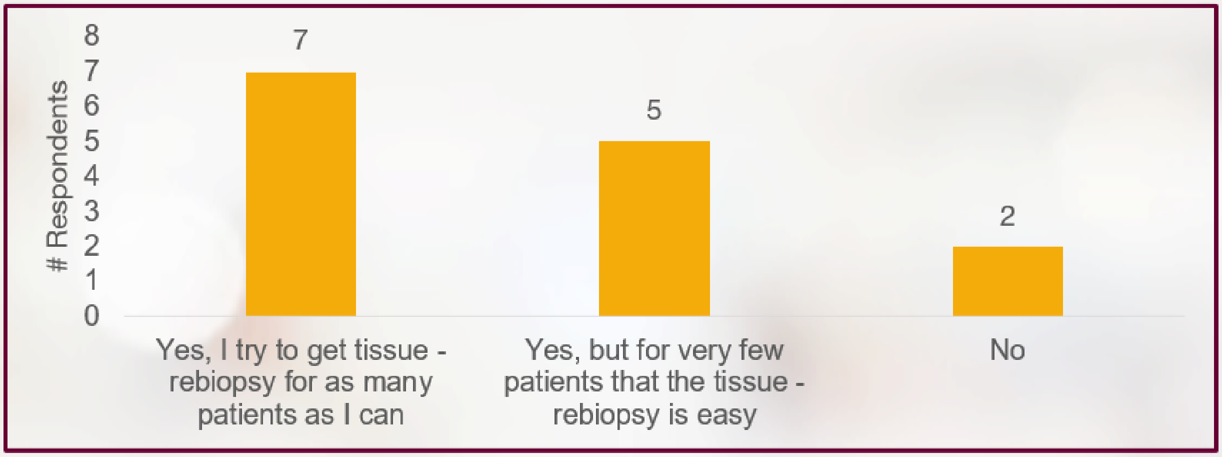

If 'No', please elaborate the reason why

Responses from 2 experts
- Chemotherapy is the only option for second line therapy (1/2)
- not all can do retissue – biopsy (1/2)

Others, please specify

- None

Q14: Please select the most significant challenge when conducting tissue rebiopsy at disease progression in stage-IV EGFR-mutated non-small-cell lung cancer (NSCLC) patients on TKI therapy.

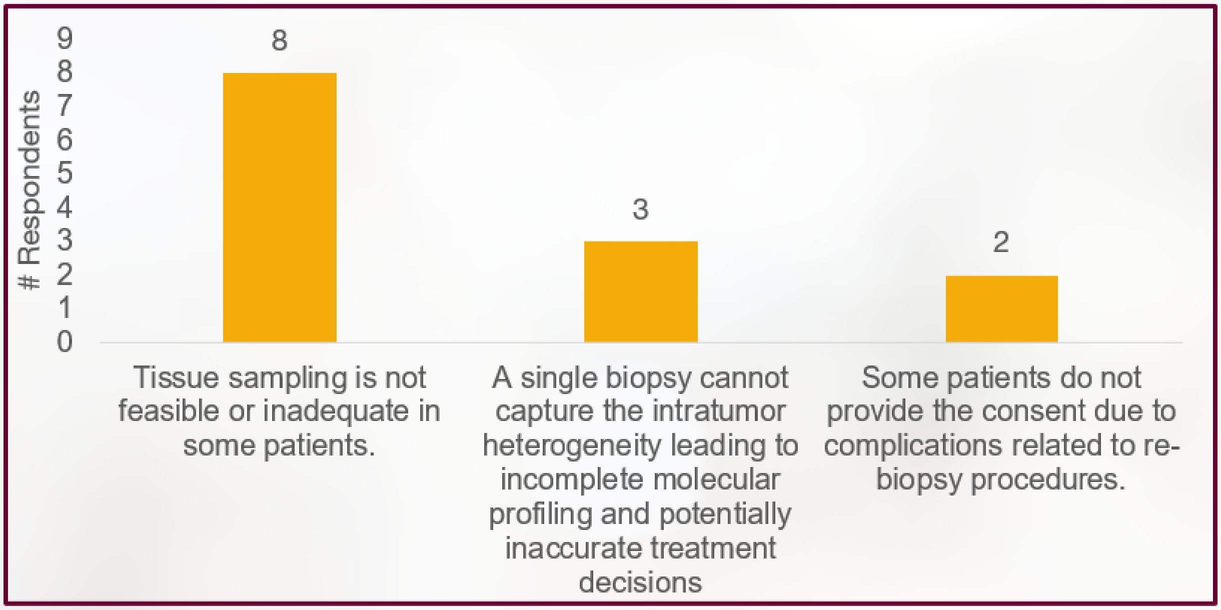

Others, please specify

Response from 1 expert

- all of above (1/1)

Q15: In your clinical practice, when the patients progressed on Osimertinib in 1L setting, which scenario/s of progression would you continue Osimertinib (alone or in combination with other treatments)?

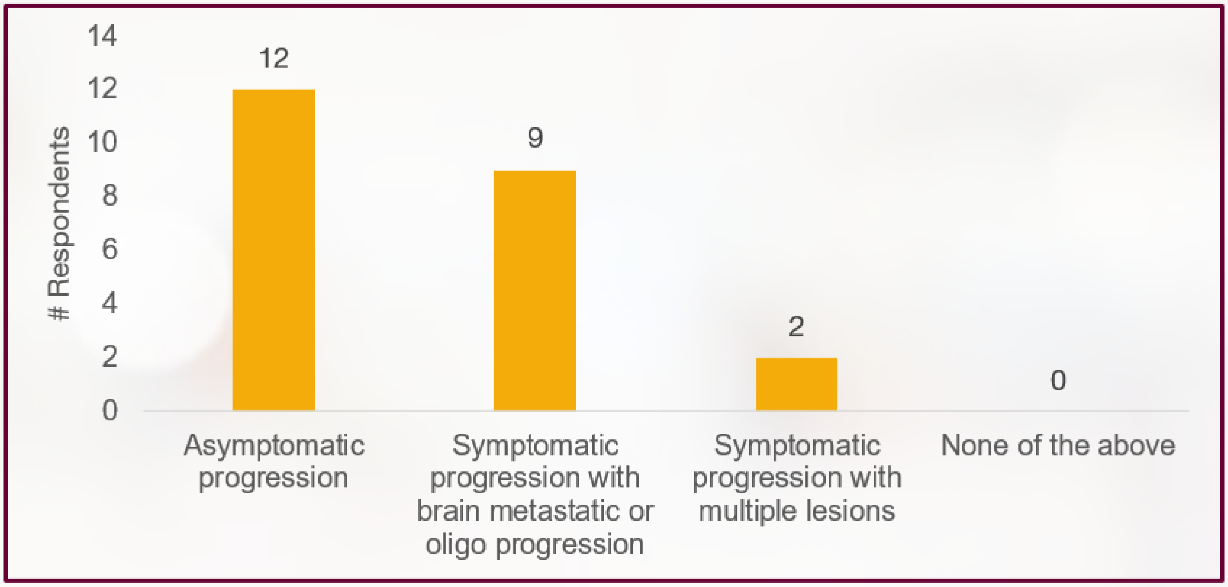

Others, please specify

Response from 1 expert

- low tumor volume (1/1)

Q16: Based on current data of TROP2-Directed ADCs, in your opinion what would be the implication of ADCs for 2L+ stage IV NSCLC patients?

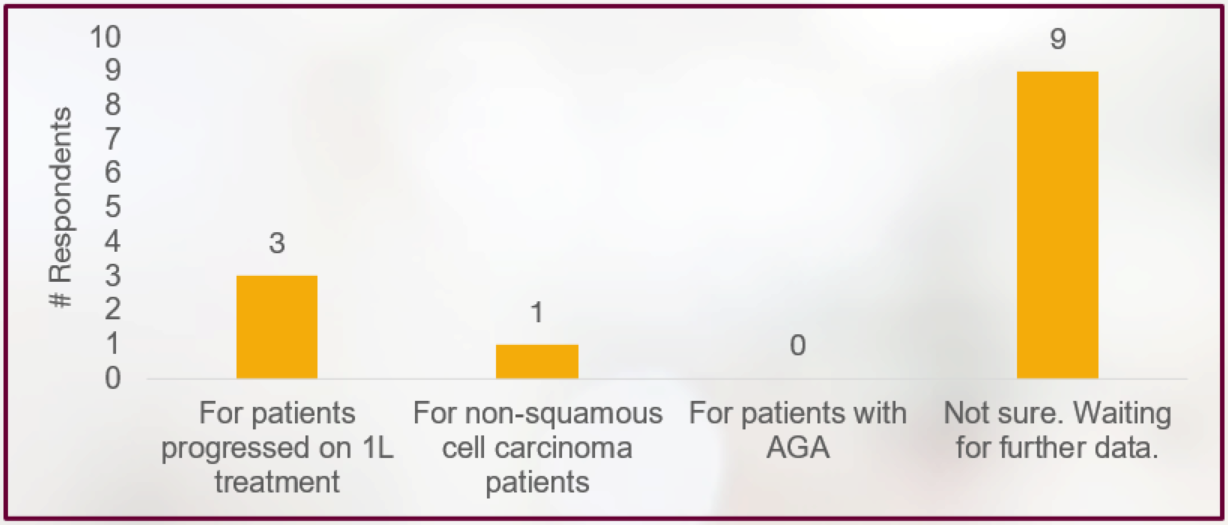

Others, please specify

Response from 1 expert

- In Heavily Pre-Treated Patients (1/1)

Q17: Do you perform MET testing for the patients who progressed on Osimertinib?

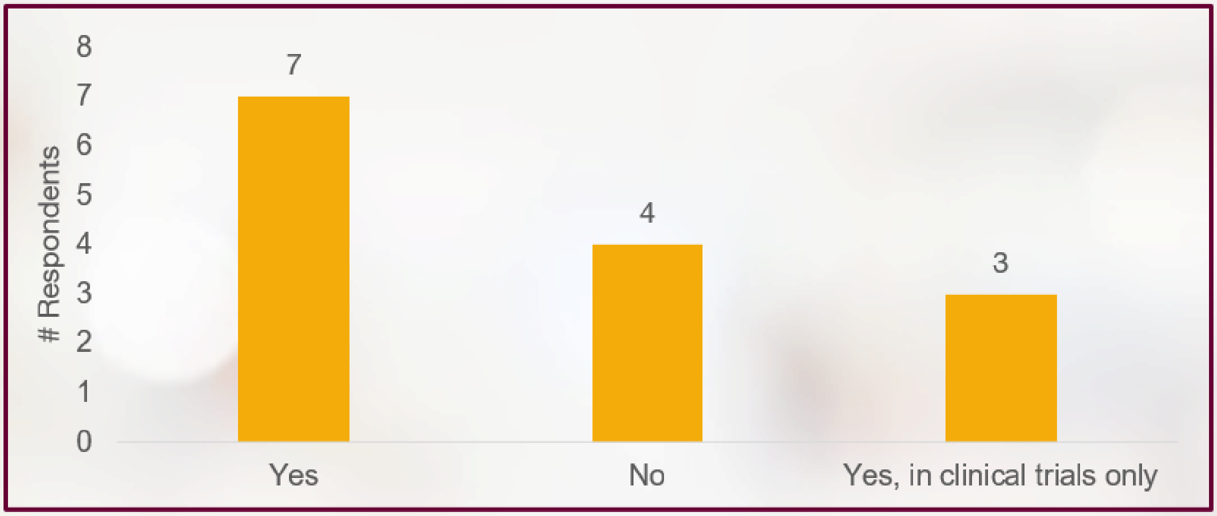

If 'Yes', Which platform and cut-offs are used?

Responses from 7 experts

- NGS/ liquid NGS in house testing (5/7)
- MET IHC 2+, or 3+ (1/7)
- FISH (1/7)
- not routinely (1/7)
- NGS with cutoff for amplification being 6 (1/7)

Voting questions and responses

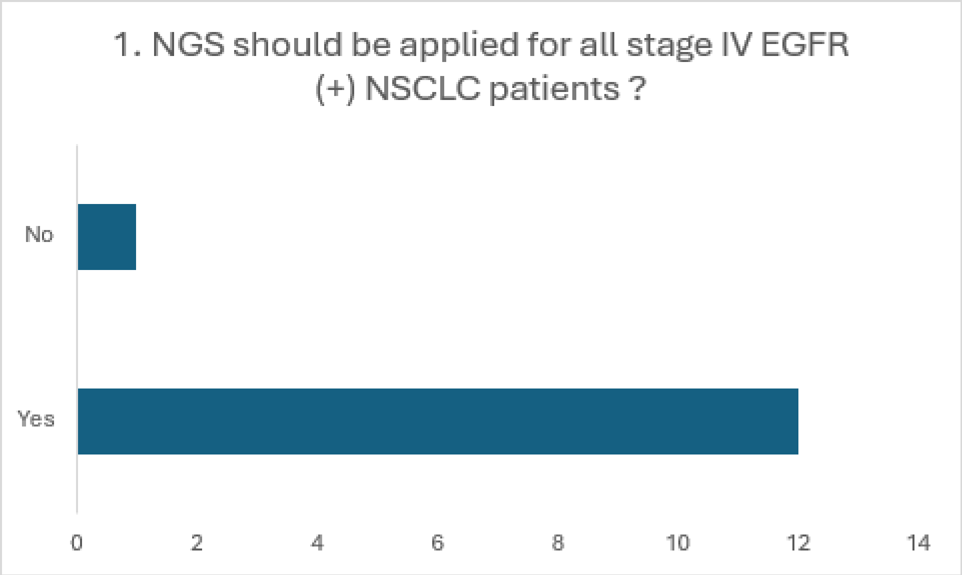

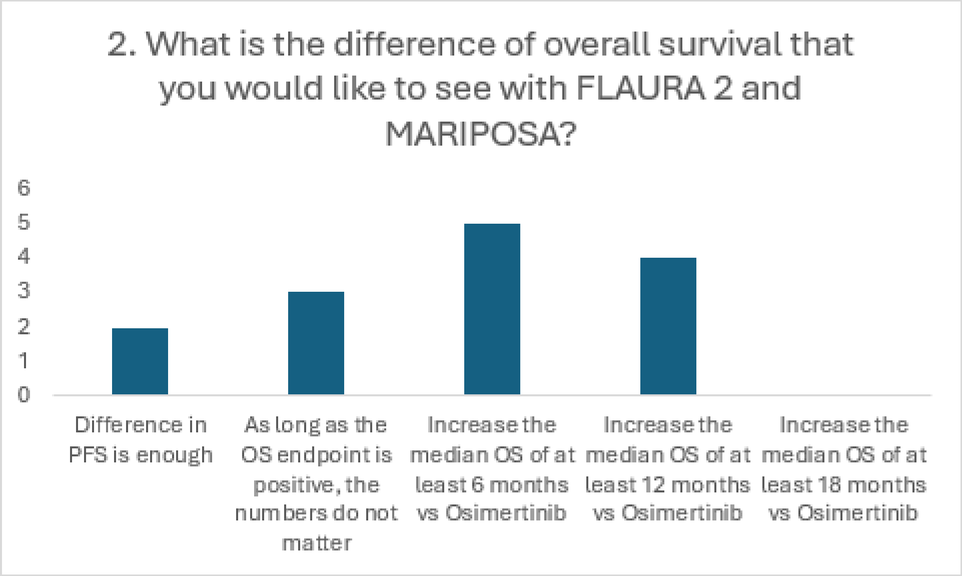

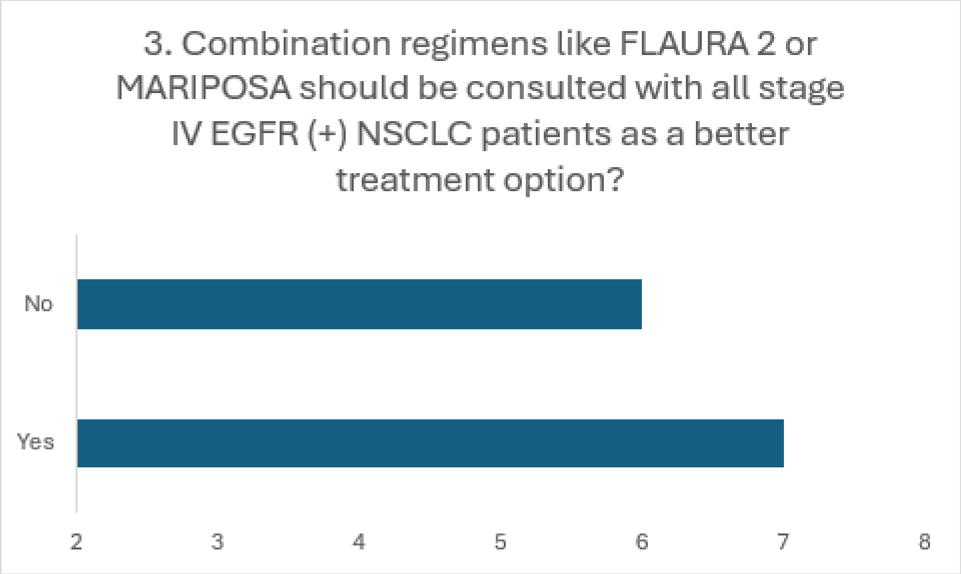
3. Combination regimens like FLAURA 2 or MARIPOSA should be consulted with all stage IV EGFR (+) NSCLC patients as a better treatment option?
No
Yes
2
3
4
5
6
7
8

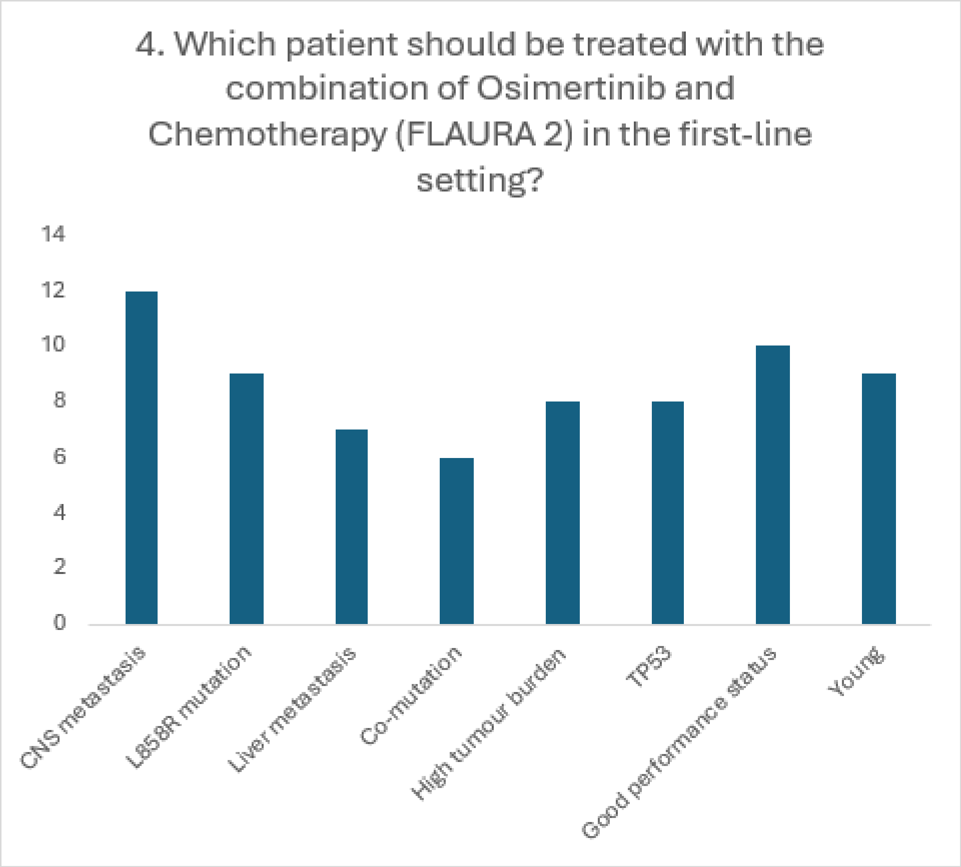
4. Which patient should be treated with the combination of Osimertinib and Chemotherapy (FLAURA 2) in the first-line setting?
14
12
10
8
6
4
2
0
CNS metastasis
L858R mutation
Liver metastasis
Co-mutation
High tumour burden
TP53
Good performance status
Young

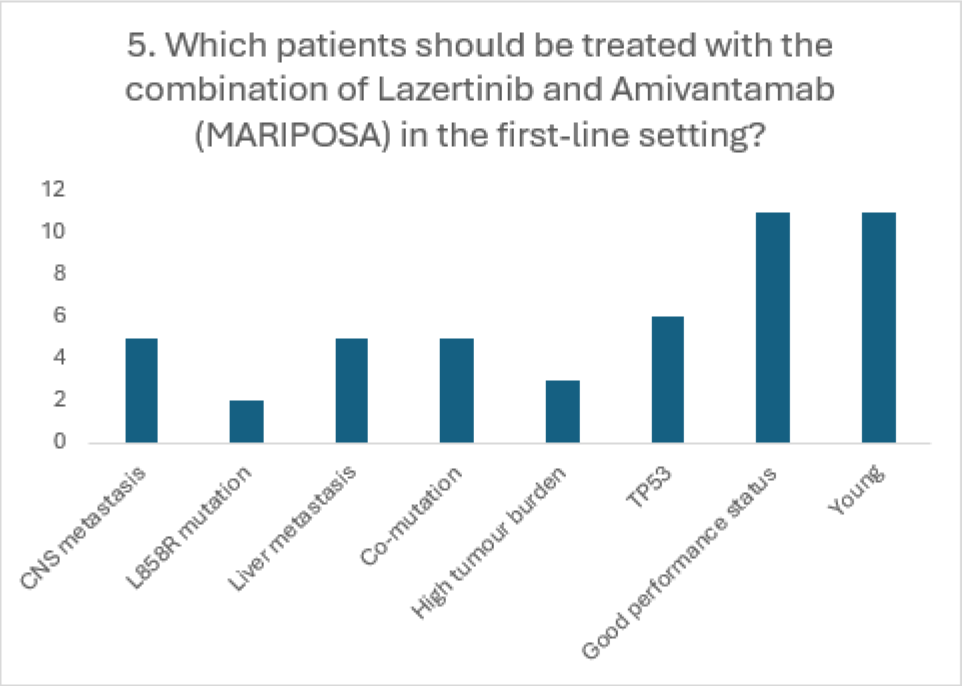
5. Which patients should be treated with the combination of Lazertinib and Amivantamab (MARIPOSA) in the first-line setting?
12
10
8
6
4
2
0
CNS metastasis
L858R mutation
Liver metastasis
Co-mutation
High tumour burden
TP53
Good performance status
Young

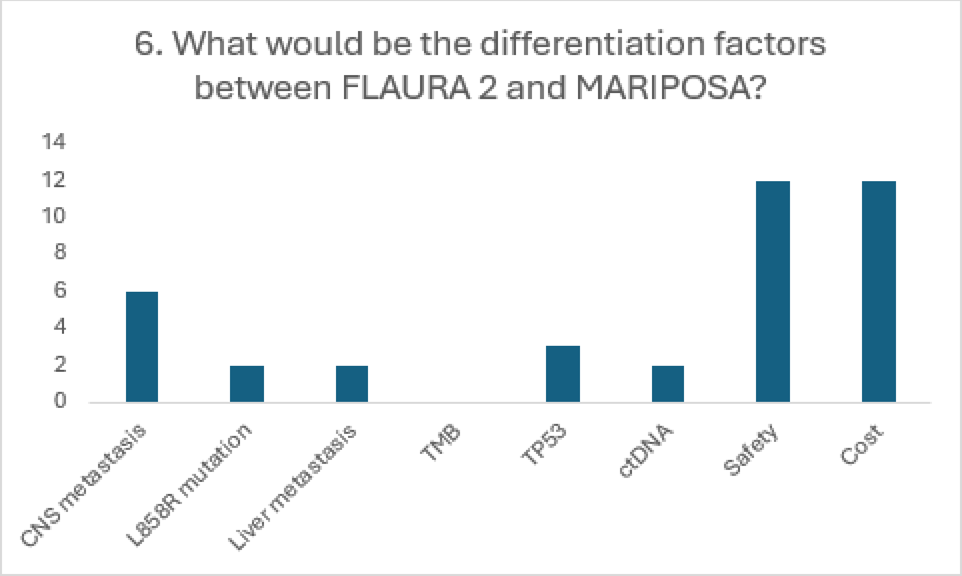
6. What would be the differentiation factors between FLAURA 2 and MARIPOSA?
14
12
10
8
6
4
2
0
CNS metastasis
L858R mutation
Liver metastasis
TMB
TP53
ctDNA
Safety
Cost

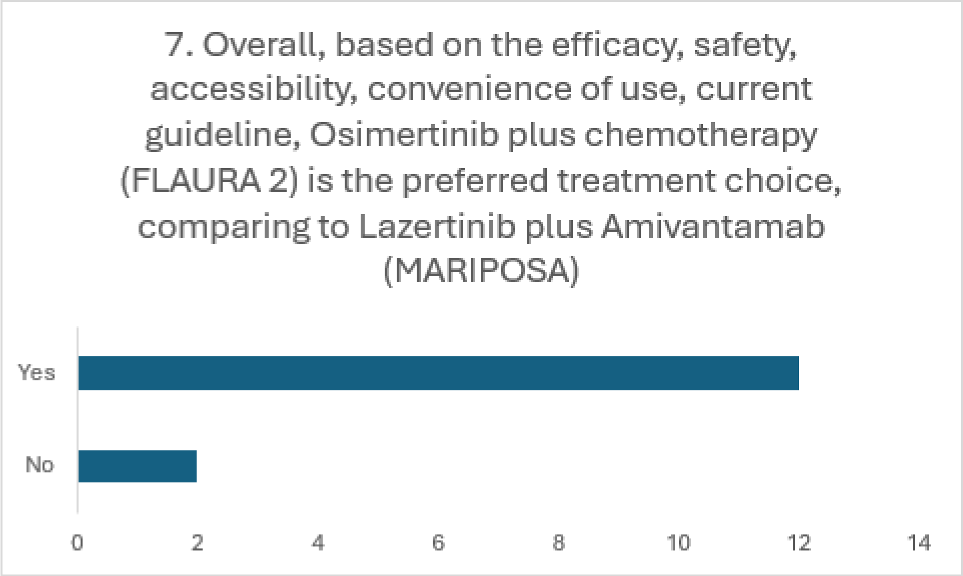
7. Overall, based on the efficacy, safety, accessibility, convenience of use, current guideline, Osimertinib plus chemotherapy (FLAURA 2) is the preferred treatment choice, comparing to Lazertinib plus Amivantamab (MARIPOSA)
Yes
No
0
2
4
6
8
10
12
14

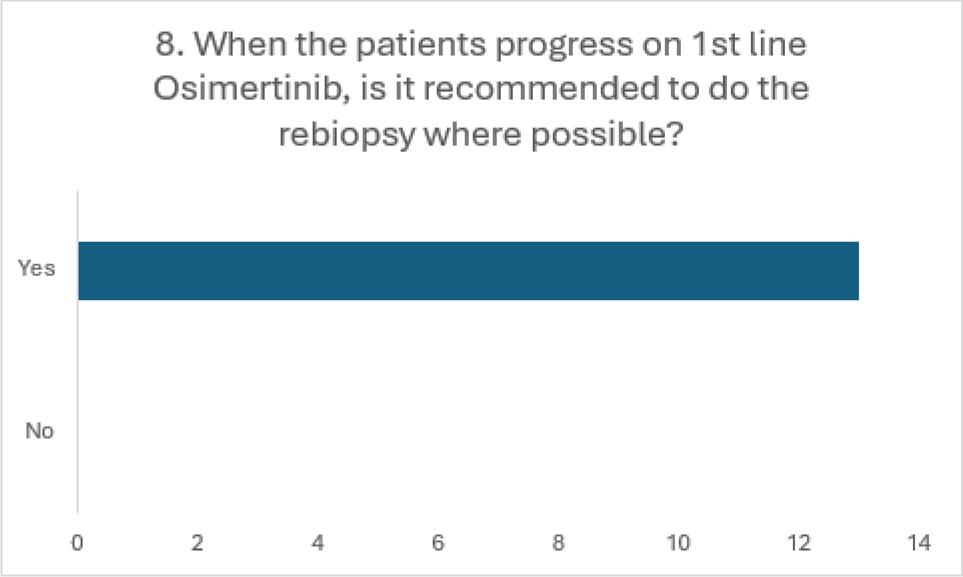
8. When the patients progress on 1st line Osimertinib, is it recommended to do the rebiopsy where possible?
Yes
No
0
2
4
6
8
10
12
14

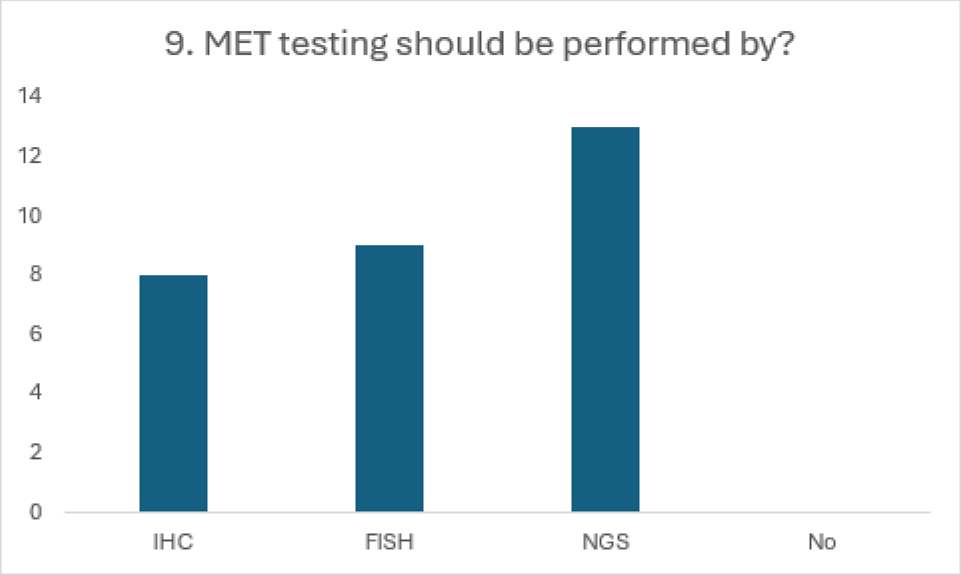
9. MET testing should be performed by?
14
12
10
8
6
4
2
0
IHC
FISH
NGS
No

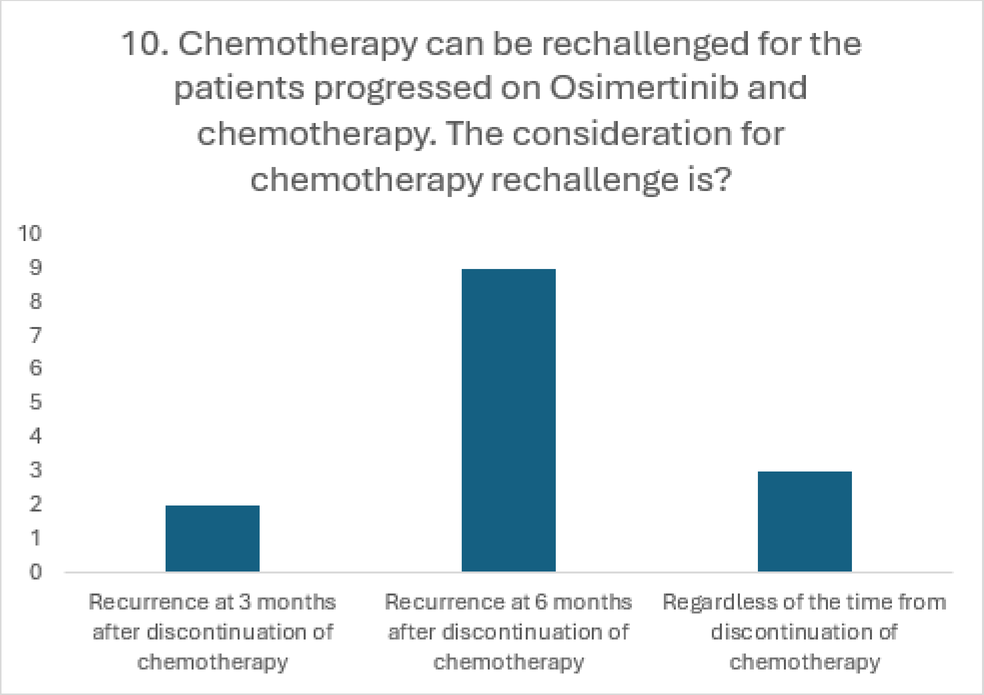
10. Chemotherapy can be rechallenged for the patients progressed on Osimertinib and chemotherapy. The consideration for chemotherapy rechallenge is?
10
9
8
7
6
5
4
3
2
1
0
Recurrence at 3 months after discontinuation of chemotherapy
Recurrence at 6 months after discontinuation of chemotherapy
Regardless of the time from discontinuation of chemotherapy
